# Patients with neurological or psychiatric complications of COVID-19 have worse long-term functional outcomes: COVID-CNS—A multicentre case–control study

**DOI:** 10.1038/s41598-024-80833-0

**Published:** 2025-01-27

**Authors:** Rajish S. K. Shil, Adam Seed, Nkongho Egbe Franklyn, Brendan F. Sargent, Greta K. Wood, Yun Huang, Katherine C. Dodd, James B. Lilleker, Thomas A. Pollak, Sylviane Defres, Thomas M. Jenkins, Nicholas W. S. Davies, David A. Cousins, Michael S. Zandi, Thomas A. Jackson, Laura A. Benjamin, Ava Easton, Tom Solomon, John R. Bradley, Patrick F. Chinnery, Craig J. Smith, Timothy R. Nicholson, Alan Carson, Rhys H. Thomas, Mark Alexander Ellul, Nicholas W. Wood, Gerome Breen, Benedict Daniel Michael, the COVID-CNS Study Group, Benedict Daniel Michael, John P. Aggleton, Ali M. Aggleton, Ammar Al-Chalabi, Christopher M. Allen, Jay Amin, Cherie Armour, Mark R. Baker, Suzanne Barret, Neil Basu, Rahul Batra, Alex Berry, Laura Benjamin, Richard A. I. Bethlehem, Bethan Blackledge, Sarah A. Boardman, John Bradley, David P. Breen, Gerome Breen, Judith Breuer, Matthew Broome, Ed Bullmore, Matthew Butler, Alan Carson, Hannah Castell, Jonathan Cavanagh, Patrick Chinnery, David Christmas, David M. Christmas, Jonathan R. I. Coleman, Alaistair Coles, Ceryce Collie, Nadine Cossette, David Cousins, Colm Cunningham, Alastair Darby, Anthony S. David, Nicholas Davies, Sylviane Defres, Katherine C. Dodd, Alex Dregan, Eugene Duff, Cordelia Dunai, Ava Easton, Franklyn N. Egbe, Mark A. Ellul, Nikos Evangelou, Bethany Facer, Peter M. Fernandes, Richard Francis, Ian Galea, Afagh Garjani, Lily George, Valentina Giunchiglia, Kiran Glen, Rebecca Gregory, Michael Griffiths, Victoria Grimbly, Alexander Grundmann, Savini Gunatilake, Shahd H. M. Hamid, Adam Hampshire, Marc Hardwick, Jade D. Harris, Ewan Harrison, NeiL A. Harrison, Paul J. Harrison, Monika Hartmann, Peter J. Hellyer, Claire Hetherington, Orla Hilton, Julian Hiscox, Eva Maria Hodel, Angela E. Holland, Matthew Hotopf, Yun Huang, Stella Hughes, Masud Husain, Sarosh Irani, Thomas A. Jackson, Thomas M. Jackson, Peter Jezzard, Johan Kallberg Zvrskovec, Gursharan Kalsi, Simon Keller, Nathalie Kingston, Sandar Kyaw, E. Charles Leek, Gabriella Lewis, James B. Lilleker, Michael P. P. Lunn, Claire L. MacIver, Daniel Madarshahian, Parisa Mansoori, Naomi Martin, Gavin McDonnell, Emily McGlinchey, Stephen McKeever, Ryan McIlwaine, Andrew M. McIntosh, David K. Menon, Benedict D. Michael, Karla L. Miller, Dina Monssen, Christopher M. Morris, Ciaran Mulholland, Akshay Nair, Edward Needham, Virginia Newcombe, Nathalie Nicholas, Timothy R. Nicholson, Ronan O’Malley, Obioma Orazulume, Marlies Ostermann, Stella-Maria Paddick, Alish Palmos, Arvind Patel, Sharon Peacock, Sophie L. Pendered, Sarah L. Pett, Thomas A. Pollak, Angela Roberts, Henry C. Rogers, Silvia Rota, Rustam Al-Shahi Salman, Merna Samuel, Brendan F. Sargent, Stephen J. Sawcer, Adam W. Seed, Scott Semple, Pamela J. Shaw, Rajish S. K. Shil, Adam Sieradzki, Bhagteshwar Singh, Craig J. Smith, Jacqueline Smith, Stephen M. Smith, Tom Solomon, Leonie Taams, Arina Tamborska, John-Paul Taylor, Kukatharmini Tharmaratnam, Rhys H. Thomas, Emma Thomson, William Trender, Zain-Ul-Abideen Ahmad, Jonathan Underwood, Rachel Upthegrove, Tonny Veenith, Annalena Venneri, Angela Vincent, Daniel j. van Wamelen, Guy Williams, Steven Williams, Sui Hsien Wong, Greta K. Wood, Nicholas Wood, Michael S. Zandi, Fernando Zelaya, Kathleen E. Stirrups, Laura Cocking, Rose Eichenberger, Debbie Clapham, Hannah Stark

**Affiliations:** 1 Clinical Infection, Microbiology & Immunology, Institute of Infection, Veterinary and Ecological Sciences, University of Liverpool, Liverpool, UK; 2 Department of Neurology, Walton Centre of Neurosurgery and Neurology, Liverpool, UK; 3 Liverpool University Hospitals NHS Foundation Trust, Liverpool, UK; 4 Nuffield Department of Clinical Neurosciences, University of Oxford, John Radcliffe Hospital, Oxford, OX3 9DU UK; 5 Department of Psychiatry, University of Oxford, Oxford, OX3 7JX UK; 6 Institute of Infection, Veterinary and Ecological Sciences, National Institute for Health and Care Research Health Protection Research Unit in Emerging and Zoonotic Infections, University of Liverpool, Liverpool, UK; 7 Manchester Centre for Clinical Neurosciences, Northern Care Alliance NHS Foundation Trust, Manchester Academic Health Science Centre, Salford, M6 8HD UK; 8 Division of Musculoskeletal & Dermatological Sciences, School of Biological Sciences, Faculty of Biology, Medicine and Health, School of Medical Sciences, University of Manchester, Manchester, UK; 9 Department of Psychosis Studies, Institute of Psychiatry, Psychology and Neuroscience, King’s College London, London, SE5 4AF UK; 10 South London and Maudsley NHS Foundation Trust, London, UK; 11 Curtin University, Kent Street, Bentley, Perth, WA 6102 Australia; 12 Sheffield Institute for Translational Neuroscience, 385a Glossop Road, Sheffield, UK; 13 Department of Neurology, Joondalup Healthcare Campus, 60 Shenton Avenue, JoondalupPerth, WA 6027 Australia; 14 Midland St John of God Hospital, 1 Clayton Street, Midland, Perth, WA 6056 Australia; 15 Department of Neurology, Charing Cross Hospital, London, UK; 16 Translational and Clinical Research Institute, Faculty of Medical Sciences, Newcastle University, Campus for Ageing and Vitality, Newcastle Upon Tyne, NE4 5PL UK; 17 UCL Queen Square Institute of Neurology, University College London, London, WC1N 3BG UK; 18 MRC-Versus Arthritis Centre for Musculoskeletal Ageing Research, Institute of Inflammation and Ageing, University of Birmingham, Birmingham, UK; 19 Laboratory of Molecular and Cell Biology, University College London, Gower St, King’s Cross, London, WC1E 6BT UK; 20 Encephalitis International, Malton, UK; 21 The Pandemic Institute, The Spine, Liverpool, L7 3FA UK; 22 NIHR BioResource, Cambridge University Hospitals NHS Foundation, Cambridge Biomedical Campus, Cambridge, UK; 23 Department of Clinical Neurosciences, University of Cambridge, Cambridge, UK; 24 MRC Mitochondrial Biology Unit, University of Cambridge, Cambridge, UK; 25 Geoffrey Jefferson Brain Research Centre, Clinical Sciences Building, Northern Care Alliance NHS Foundation Trust, Salford, M6 8FJ UK; 26 Division of Cardiovascular Sciences, Faculty of Biology, Medicine and Health, School of Medical Sciences, University of Manchester, Manchester, UK; 27 Centre for Clinical Brain Sciences, University of Edinburgh, Edinburgh, EH16 4SB UK; 28 Translational and Clinical Research, Newcastle University, Newcastle, NE1 7RU UK; 29 Wellcome Centre for Mitochondrial Research, Newcastle University, Newcastle, NE2 4HH UK; 30 Department of Neurology, Royal Victoria Infirmary, Newcastle, NE1 4LP UK; 31 Department of Molecular Neuroscience, UCL Institute of Neurology, London, UK; 32 School of Psychology, Cardiff University, Cardiff, UK; 33 King’s College London, London, UK; 34 University of Nottingham, Nottingham, UK; 35 University of Southampton, Southampton, UK; 36 Queen’s University Belfast, Belfast, UK; 37 Newcastle University, Newcastle, UK; 38 Northern Health and Social Care Trust, Antrim, UK; 39 University of Glasgow, Glasgow, UK; 40 Department of Psychology, University of Cambridge, Cambridge, UK; 41 Salford Royal, Northern Care Alliance NHS Foundation Trust, Manchester, UK; 42 University College London, London, UK; 43 Institute for Mental Health, School of Psychology, University of Birmingham, Birmingham, UK; 44 University of Dundee, Dundee, UK; 45 Royal Infirmary of Edinburgh, NHS Lothian, Edinburgh, UK; 46 School of Biochemistry and Immunology, Trinity Biomedical Sciences Institute, Trinity College Dublin, Dublin, Ireland; 47 University of Liverpool, Liverpool, UK; 48 Department of Psychiatry, Institute of Mental Health, UCL, Liverpool, UK; 49 UK Dementia Research Institute, Department of Brain Sciences, Imperial College London, London, UK; 50 Department of Pharmacology and Therapeutics, Institute of Systems, Molecular and Integrative Biology, University of Liverpool, Liverpool, UK; 51 Centre for Clinical Brain Sciences, University of Edinburgh, Edinburgh, UK; 52 The Stroke Association, London, UK; 53 Imperial College London, London, UK; 54 Sheffield Teaching Hospitals NHS Foundation Trust, Sheffield, UK; 55 Royal Stoke University Hospital, Stoke-On-Trent, UK; 56 Department of Neuroimaging, Institute of Psychiatry, Psychology & Neuroscience, King’s College London, London, UK; 57 Salford Royal, Northern Care Alliance NHS Foundation Trust, Manchester, UK; 58 University of Cambridge, Cambridge, UK; 59 Cardiff University Brain Research Imaging Centre, School of Medicine, Cardiff University, Cardiff, UK; 60 Department of Psychiatry, Warneford Hospital, University of Oxford, Oxford, UK; 61 Nottingham University Hospital, Nottingham, UK; 62 Belfast Health and Social Care Trust, Belfast, UK; 63 Social, Genetic and Developmental Psychiatry Centre, Institute of Psychiatry, Psychology & Neuroscience, King’s College London, London, UK; 64 Institute of Mental Health, Nottingham, UK; 65 Cardiff University, Cardiff, UK; 66 National Institute for Health Research (NIHR) Bioresource, London, UK; 67 MRC Clinical Trials Unit, UCL, London, UK; 68 Institute of Clinical Trials and Methodology, UCL, London, UK; 69 Department of Health Data Science, Institute of Population Health, University of Liverpool, Liverpool, UK; 70 University of Sheffield, Sheffield, UK; 71 COVID-CNS Consortium, Liverpool, UK; 72 NIHR Bioresource, Cambridge Biomedical Campus, Cambridge, UK; 73 Department of Haematology, University of Cambridge, Cambridge Biomedical Campus, Cambridge, UK

**Keywords:** Central nervous system infections, Stroke

## Abstract

It is established that patients hospitalised with COVID-19 often have ongoing morbidity affecting activity of daily living (ADL), employment, and mental health. However, little is known about the relative outcomes in patients with COVID-19 neurological or psychiatric complications. We conducted a UK multicentre case–control study of patients hospitalised with COVID-19 (controls) and those who developed COVID-19 associated acute neurological or psychiatric complications (cases). Among the 651 patients, [362 (55%) cases and 289 (45%) controls], a higher proportion of cases had impairment in ADLs (199 [68.9%] vs 101 [51.8%], OR 2.06, p < 0.0002) and reported symptoms impacting employment (159 [58.2%] vs 69 [35.6%] OR 2.53, p < 0.0001). There was no significant difference in the proportion with depression or anxiety between case and control groups overall. For cases, impairment of ADLs was associated with increased risk in female sex, age > 50 years and hypertension (OR 5.43, p < 0.003, 3.11, p = 0.02, 3.66, p = 0.04). Those receiving either statins or angiotensin converting enzyme (ACE) inhibitors had a lower risk of impairment in ADLs (OR 0.09, p = 0.0006, 0.17, p = 0.03). Patients with neurological or psychiatric complications of COVID-19 had worse functional outcomes than those with respiratory COVID-19 alone in terms of ADLs and employment. Female sex, age > 50 years, and hypertension were associated with worse outcomes, and statins or ACE inhibitors with better outcomes.

## Introduction

There is established evidence that coronavirus disease 2019 (COVID-19) is associated with a wide spectrum of acute neurological and psychiatric complications^1–3^. COVID-19-related neurological syndromes vary from mild self-reported symptoms such as headaches, myalgia, anosmia, and ageusia/dysgeusia to more severe clinical syndromes such as cerebrovascular disease, encephalopathy/delirium, inflammatory diagnoses (e.g., acute disseminated encephalomyelitis), and onset of new psychiatric diagnoses (e.g., psychosis)^1–3^. Several studies have reported high rates of acute morbidity and mortality in patients with COVID-19-associated neurological and psychiatric complications compared to the general COVID-19 population^4–7^. However, the post-acute impact of these complications on independence for activities of daily living (ADLs), return to employment, and the impact on mental health, are not well understood, particularly relative to having been hospitalised with COVID-19 more generally.

In this case–control study, we evaluated patient-centred functional outcomes after discharge from hospital in the form of impact on their ADLs, if these symptoms impacted their employment, and assessed this relative to mental health measures. This was performed to identify the proportions of patients affected and risk factors associated with poor outcomes, so that early and appropriate rehabilitation and support could be provided to those affected in order to prevent longer-term morbidities.

## Aims and objectives

1. Assess the post-acute functional outcomes of patients discharged from the hospital with a neurological or psychiatric complication of COVID-19 relative to patients hospitalised with COVID-19 without these complications.
2. Identify risk factors associated with poor functional outcomes in patients with COVID-19-related neurological or psychiatric complications relative to patients hospitalised with COVID-19 without these complications.
3. Assess the proportion of these patients (cases/controls) having symptoms affecting their employment, and also to assess anxiety and depression in these two groups.

## Methodology

### Study design

Hospitalised adult patients (aged > 16yrs) were recruited into the COVID Clinical Neuroscience Study (COVID-CNS) in the UK if they met the WHO criteria for definite or probable COVID-19^8^. Cases were defined as those who developed a new neurological or psychiatric diagnosis in association with COVID-19 and were classified by specific diagnostic criteria as per established Clinical Case Definitions^1,9^ [Table s1]. Where there was uncertainty in the primary diagnostic category, cases were discussed by a national multi-disciplinary team of experts in neurology, neurological infection, neuroimmunology, and psychiatry. Controls were defined as those without new neurological or psychiatric diagnoses, and control recruitment was targeted to match the cohort of cases for age, sex, premorbid Rockwood clinical frailty score, and epoch of the COVID-19 pandemic in the UK^10^. Patients with prior clinically significant neurological and/or psychiatric diagnoses were excluded (e.g., multiple sclerosis, dementia, or ICD-10 major depression). Cases with a new neurological complication of COVID-19 unrelated to previous neurological disorders were not excluded (e.g., a new diagnosis of COVID-19 myelitis in a patient with a history of a transient ischaemic attack). Cases of neurological complications from SARS-CoV-2 vaccination were excluded from this sub-study. For all participants, data were entered in the central COVID-CNS database on a standardised neurological case record form, including demographics, pre-existing conditions, frailty (Rockwood Clinical Frailty Score), and clinical details from three intervals: on admission, the nadir of the admission, and on discharge.

Participants were followed up after discharge at a median interval of 13–16 months for a structured assessment in a single face-to-face appointment to assess changes from pre-admission status with regards to function, occupational impact, and psychological symptoms using validated measures of anxiety and depression [Generalised anxiety disorder – 7 (GAD-7), Patient health questionnaire – 9 (PHQ-9)], alongside patient-reported symptoms (drawn from Amyotrophic lateral sclerosis Functional rating scale—ALSFRS, Unified Parkinson’s disease rating scale—UPDRS scales) and employment^11–15^. When patients could not attend, they were supported to complete the questionnaire online and over the telephone following discharge from the hospital.

**Fig. 1:**
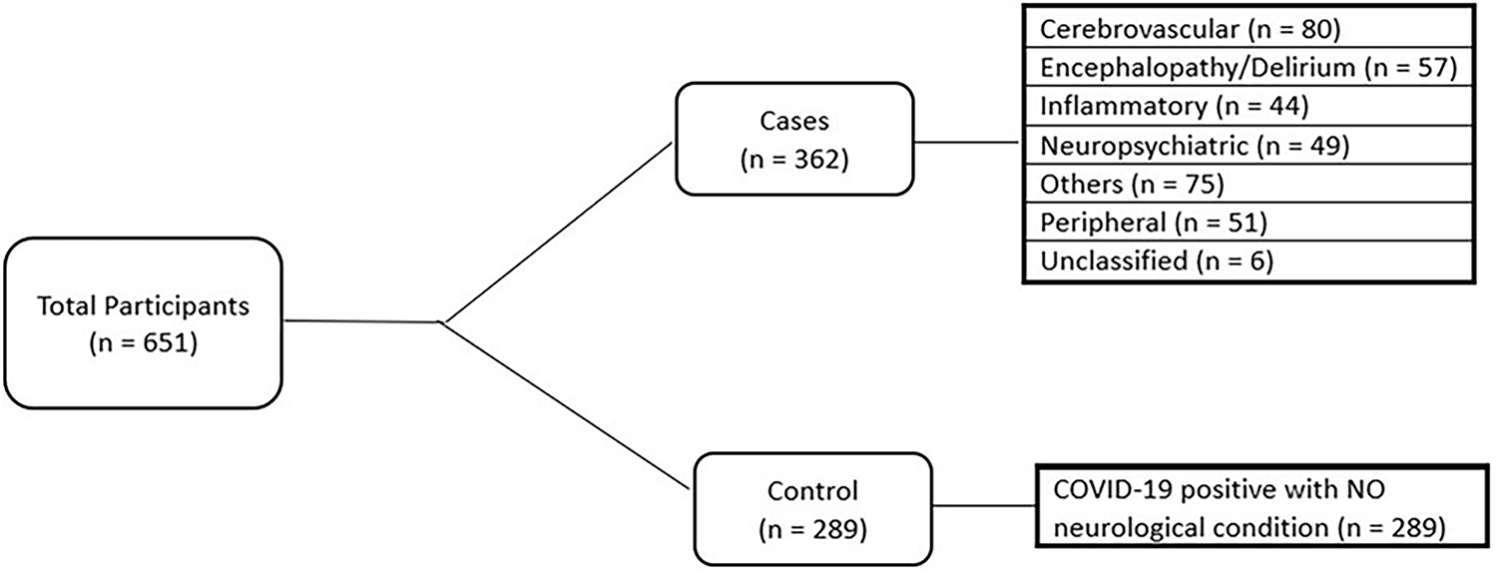
Recruitment flowchart.

A total of 651 patients, admitted to hospital between March 2020 and July 2022, were identified for follow-up who met the inclusion criteria, of whom 362 (55%) fulfilled the criteria for cases and 289 (45%) patients for controls. The cases were classified as cerebrovascular events (n = 80), encephalopathy/delirium (n = 57), peripheral neuropathies (n = 51), neuropsychiatric complications (n = 49), central inflammatory conditions (n = 44), others (n = 75) and unclassified (n = 6) [Fig. 1]. The case group “others” corresponds to patients who do not fall into a broad classified case definition group, but are a mixed group of symptoms, including those with generalised weakness, seizures, movement disorders, speech or swallowing disturbance, autonomic disturbances, headaches, anosmia, fatigue, and cerebral hypoxic injury. The median (IQR) age of cases and controls were comparable, 57 (44–64) and 56 (46–65) years respectively (p = 0.91) and there was a similar sex distribution between cases and controls as 219 (60.5%) and 158 (54.6%) were male respectively (p = 0.15) [Table 1]. However, of 651 patients recruited, follow-up data for assessment of functional outcomes was only available for 484. There were no significant differences between baseline demographic features between cases and controls [Table 1]. The specific demographics reviewed are age, sex, ethnicity, education, employment/retirement status, smoking status.

**Table 1:**
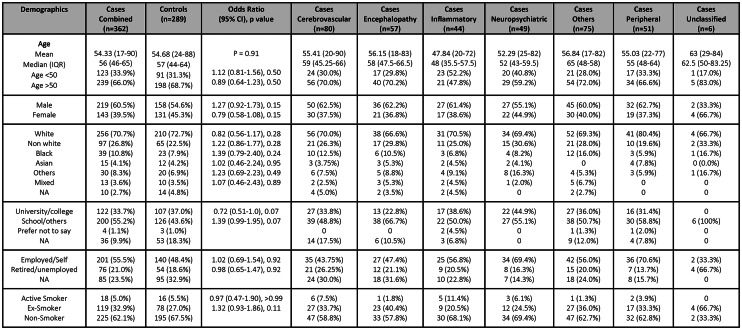
Case vs control—Demographics (see the supplementary material for further details).

IQR: Interquartile range, NA: Not Available (data), CI: Confidence intervals.

### Statistical analysis and outcome variables

The functional outcome was measured with an individualised scoring system, from responses to ADL questions (UPDRS and ALS scales). The scoring system was established from the patients’ responses to questions—difficulties in getting out of bed/chair/car, balance, walking, reading/writing, changes in hobbies, and personal hygiene. We prospectively formulated a 5-point scale [Figure s1] for these five essential domains (Normal 0, slight 1–4, mild 5–8, moderate 9–13, and severe 14–20 impairment), and with the view of sample size and descriptive statistic findings on the available data, we have dichotomised the outcome as either ADL not impaired (Score 0) or impaired (Score 1–20) for univariate and multivariate analysis, and regression modelling. This model of ADL score corresponds to the standard modified Rankin Scale (mRS), in which a normal ADL score would be equivalent to mRS 0–1, as there is no disability, and an impaired ADL score would be equivalent to mRS 2–4, as none of the participants were in a persistent vegetative state or dead to be classed within mRS 5–6 ^16^.

The ADL questions have been included as supplementary material. We have acknowledged both the rationale and limitations of this composite tool used in our study. Specifically, we did not use a single tool, but brought together components from several validated tools. The rational for this was twofold. Firstly, as the pandemic was developing, it was unclear exactly what the nature of the complications and subsequent disability would be, therefore a broad net had to be cast to incorporate potential fields of disability (including UPDRs, ALSFRS, Modified Rankin scale—mRS). Secondly, the feedback from our patient and public involvement (PPI) panel was that patients would be significantly impaired in their ability to complete long follow-up assessment sessions. We piloted this follow-up in the first 50 patients and the session took between 2-5 h depending on physical and cognitive disability and it was concluded by our PPI panel, and confirmed by informal feedback from the first 50, that this was the maximum duration which could be requested of the patients (https://www.liverpool.ac.uk/covid-clinical-neuroscience-study/patient-and-public-involvement/).

With regards to validity of this composite tool, the Cronbach’s alpha test was 0.791 and 0.777 on cases and control groups [Figure s2], indicating that the composite tool as ‘acceptable’ to assess ADL impairment^17^. In regard to anxiety and depression, anyone with GAD-7 or PHQ-9 result score > 5 is considered as having anxiety and depressive symptoms^11,13^.

To assess for risk factors associated with individual outcomes, univariate analysis was performed on demographics, medical comorbidities (hypertension, diabetes, dyslipidaemia, renal disease), admission medications including statins, angiotensin converting enzyme inhibitors (ACEi), angiotensin receptor blockers (ARB), WHO-grade COVID-19 severity reflecting oxygen requirements on admission and peak of admission, critical care admission, and inflammatory markers on admission (C-reactive protein > 5). The risk factors were extracted from the neurological case record forms of the COVID-CNS database, which were entered by the research team (Research assistants, Research nurses and associate primary investigators) from clinical hospital records. These risk factors were included in multivariate logistic regression models to estimate the odds ratio (OR) with 95% Confidence Intervals (CIs) for ADL impairment and symptoms impacting employment. The assessment of the OR for univariate and multivariate analysis was performed on the available data in the study group, excluding those with no available data. Continuous variables were analysed using a two-tailed t-test or Mann–Whitney-U test if parametric or nonparametric respectively, and the chi-squared test was used for categorical variables. Multivariate analysis and modelling were performed using logistic regression and values of p < 0.05 were considered significant (RStudio Version 2023.03.0 + 386). Multiple testing were performed for multivariate analysis, initially included all the risk factors, and then have performed a backward stepwise regression to find the best model with the highest AUROC. We have used Fisher’s exact testing for the distribution of the sample size, which showed no significant differences between the groups [Figure s3].

### Ethical approval

The COVID Clinical Neurosciences Study (COVID-CNS) was funded by the medical research council (MRC), embedded within the national NIHR BioResource, and received Research Ethics Committee approval for clinical notes review and longitudinal follow-up (REC 22/EE/0230; IRAS 313,104; HTA 12,315). All methods from data collection to analysis were carried out in accordance with relevant guidelines and regulations. Informed consent was obtained from all participants enrolled into the COVID-CNS study.

## Results

A higher proportion of cases overall had moderate and fewer had severe WHO-grade COVID-19 on admission compared to controls (OR [95%CI] 1.65 [1.2–2.29], p = 0.002 and 0.39 [0.28–0.54], p < 0.0001, respectively) [Table 2]. However, there was a greater proportion of cases requiring ventilation on admission than controls, suggesting a dichotomy of disease severity in the case group (OR [95%CI] 1.80 [1.21–2.70], p = 0.003). Nevertheless, ultimately, cases were more likely than controls to require ventilation and critical care support during the peak of admission (OR [95%CI] 1.65 [1.15–2.37], p = 0.006 and 2.50 [1.74–3.65], p = 0.0001 respectively). There was no significant difference in the pre-admission proportion with an abnormal Clinical Frailty Score, elevated BMI, or any prior neurological or psychiatric diagnoses, between cases and controls overall. Cases were more likely to have had a prior cerebrovascular disease, although the numbers were small (n = 13) and this was unrelated to their acute neurological COVID-19 complication (cerebrovascular events [n = 7], encephalopathy [n = 2], inflammatory [n = 1], and others [n = 3]).

**Table 2:**
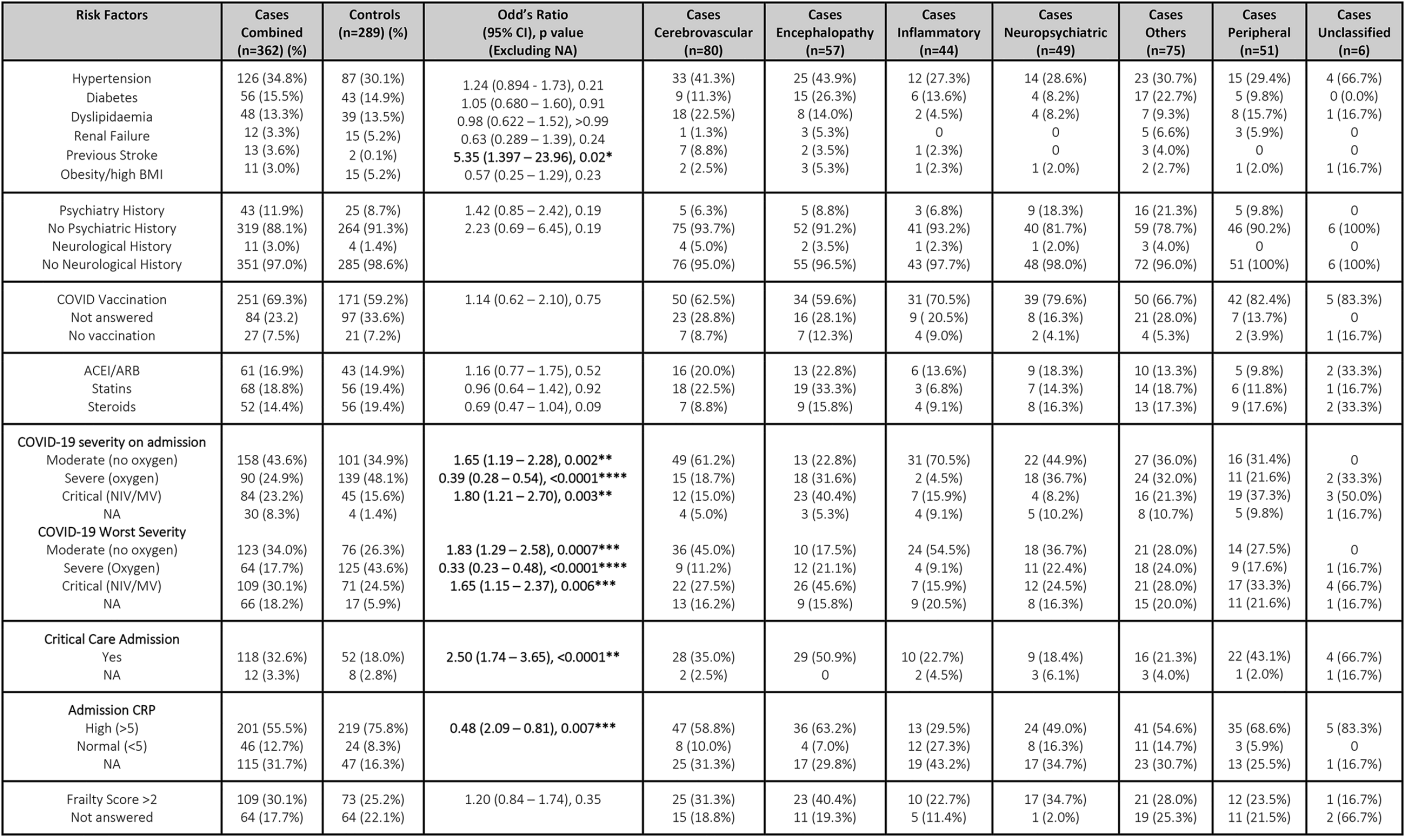
Case vs control—Clinical features (see the supplementary material for further details).

NA: Not Available (data), CI: Confidence intervals, BMI: Body mass index, ACEI: Angiotensin converting enzyme inhibitors, ARB: Angiotensin receptor blockers, NIV: Non invasive ventilation, MV: Mechanical ventilation, CRP: C-reactive protein.

**Fig. 2:**
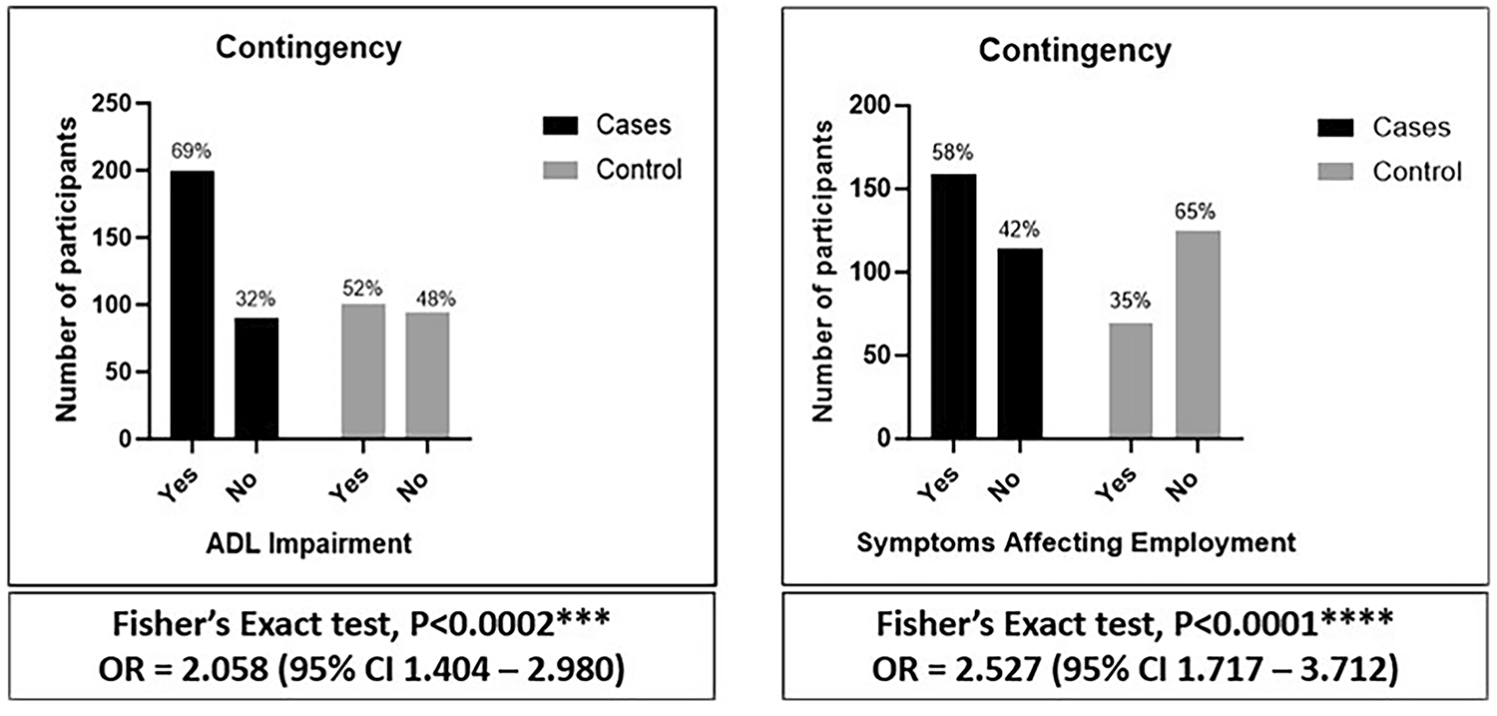
Cases vs controls, ADL and Employment.

**Fig. 3:**
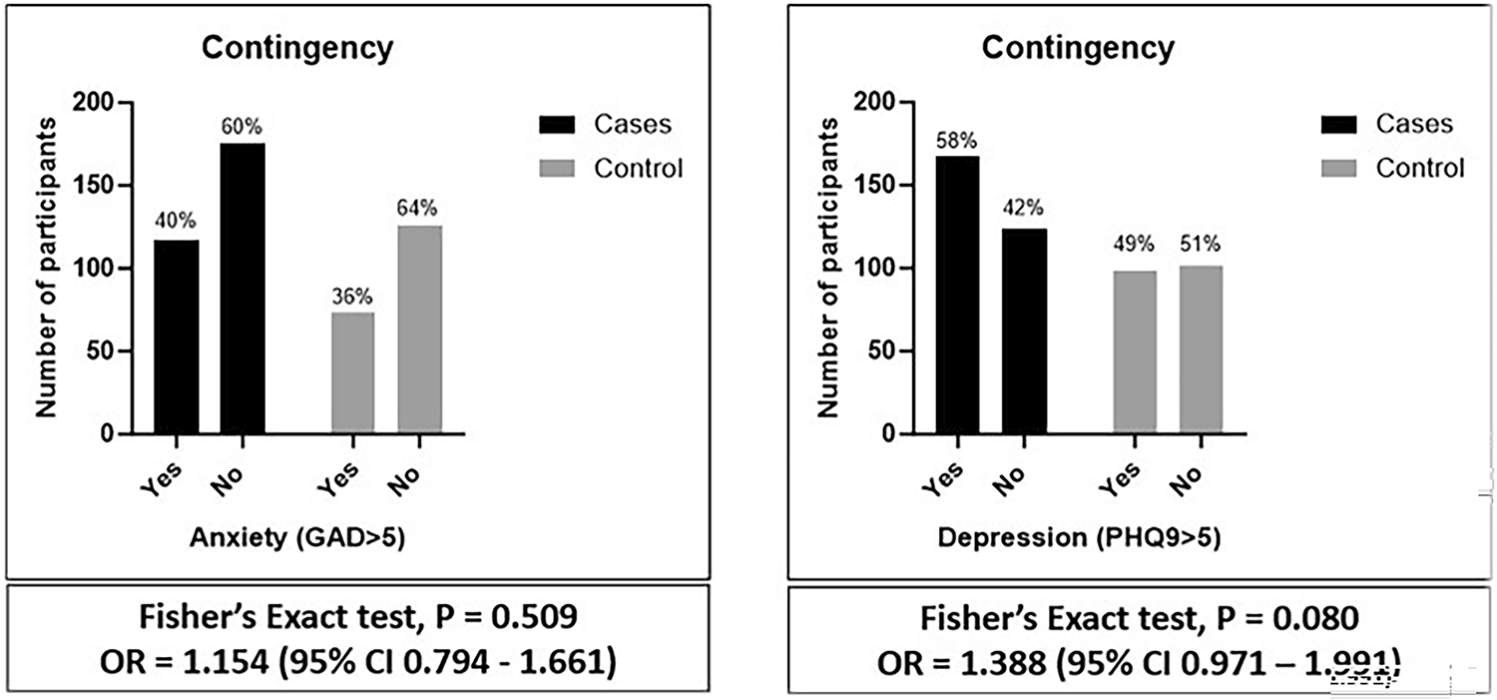
Cases vs Controls, anxiety and depression.

A higher proportion of cases compared to controls had impairment in ADLs at follow-up at the median interval of 13–16 months (199/289 [68.9%] vs 101/195 [51.8%] respectively, OR [95%CI] 2.06 [1.4–2.98], p < 0.0002) [Table 3], [Fig. 2]. Cases were also more likely than controls to report symptoms that impacted employment than controls (159/273 (58.2%) vs 69/194 [35.6%] respectively, OR [95%CI] 2.53 [1.72–3.71], p < 0.0001), and a higher percentage had become unemployed following discharge (34 [9.4%] vs 12 [4.2%] respectively). There was no significant difference in the time from discharge to completing the follow-up assessment at a median interval of 13–16 months [Table 3], or the proportion with GAD-7 or PHQ-9 scores > 5 between cases and controls overall. Within specific diagnostic groups, the greatest proportion who had impairment in ADLs relative to controls were those who had had a neuropsychiatric or peripheral complication (OR [95%CI] 2.4 [1.14–4.85], p = 0.01 and 3.72 [1.75–8.25], p = 0.0007, respectively) [Table 3]. The greatest proportions with symptoms impacting employment were those with neuropsychiatric, inflammatory, encephalopathy or peripheral complications (OR [95%CI] 4.18 [2.0–8.24], p < 0.0001; 3.26 [1.40–7.31], p0.006; 2.45 [1.25–4.72], p = 0.01; and 3.38 [1.66–6.7], p = 0.0005, respectively). There was a significantly higher proportion of patients with PHQ-9 scores >5 at follow-up for those who had had encephalopathy or a neuropsychiatric complication (OR [95%CI] 2.38 [1.16–4.74], p = 0.01 and 2.06 [1.04–3.99], p = 0.03 respectively). However, there were no significant differences in the proportions with GAD-7 scores >5 between any of the diagnostic groups of cases and controls [Fig. 3].

**Table 3:**
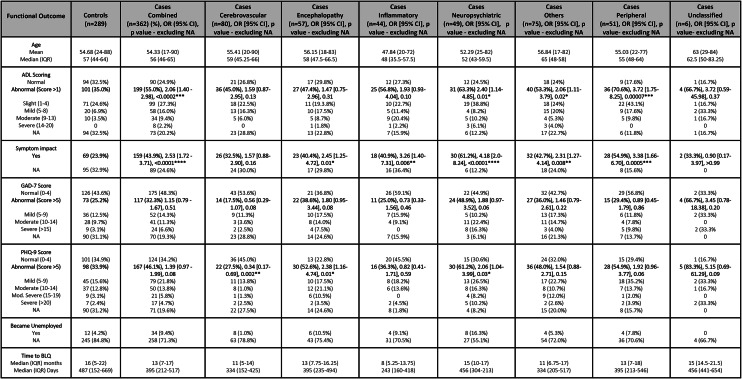
Controls vs control and individual case definitions—Functional outcomes (see the supplementary material for further details).

IQR: Interquartile range, NA: Not Available (data), OR: Odds ratio, CI: Confidence intervals, ADL: Activities of daily living, GAD-7: Generalised Anxiety Disorder – 7, PHQ-9: Patient Health Questionnaire – 9, BLQ: Baseline Questionnaire.

**Fig. 4:**
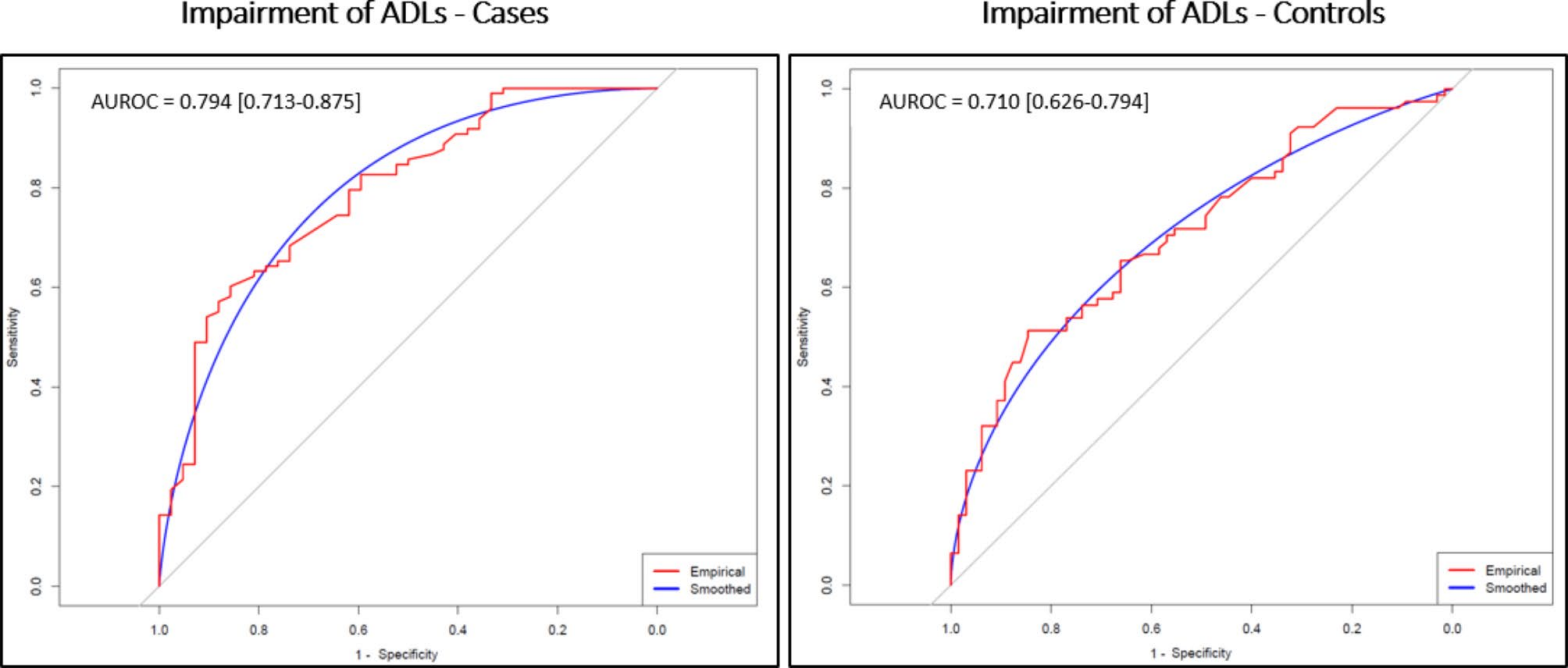
Cases and Controls—Risk factors for ADL impairment ROC curve.

**Fig. 5:**
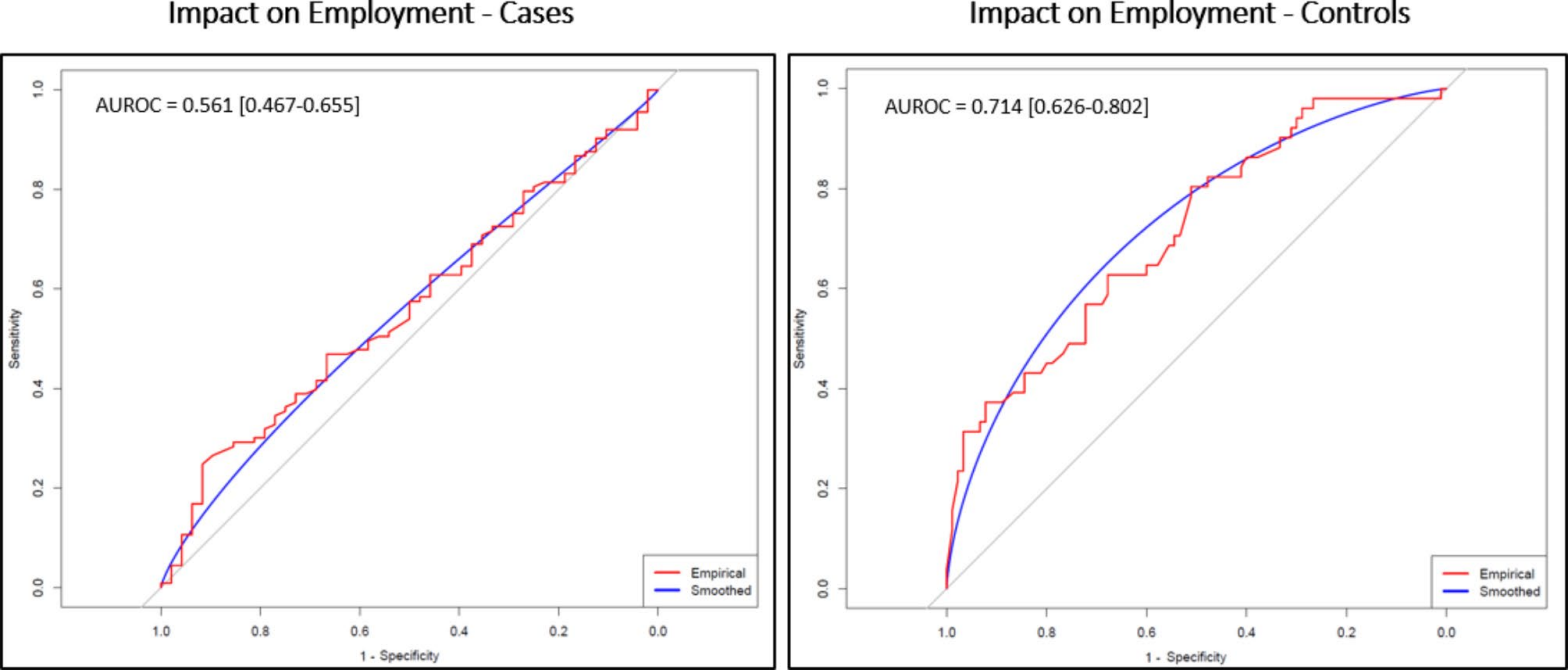
Cases and Controls—Risk factors for impact on employment ROC curve.

Among the case group with impaired ADLs, univariate analyses identified smoking (current or previous smoker) as a risk factor (OR [95% CI] 1.80 [1.05–3.14], p = 0.03) and use of angiotensin-converting enzyme inhibitors or angiotensin receptor blockers as a protective factor (OR [95% CI] 0.46 [0.23–0.94], p = 0.03) [Table 4]. On multivariate analysis for cases, impairment of ADLs was associated with increased risk in females, those aged > 50yrs, and known medical history of hypertension (OR [95%CI] 5.43 [1.79–16.96], p = 0.003, 3.11 [1.17–8.26], p = 0.02, 3.67 [1.06–12.68], p = 0.04 respectively). Those cases who were receiving either statins or angiotensin inhibiting medication on admission had a lower risk of impairment in ADLs at follow-up (OR [95%CI] 0.09 [0.02–0.36], p  =0.0006 and 0.17 [0.03–0.84], p = 0.03 respectively). In the multivariate model, using these parameters the AUROC [95%CI] was 0.794 [0.713–0.875] [Fig. 4], [Table 4]. For controls, multivariate analysis identified an increased risk of impairment of ADLs at follow-up for females only (OR [95%CI] 2.36 [1.083–5.137], p < 0.01) and the AUROC [95%CI] was 0.710 [0.626–0.794] [Fig. 4]. With regards to employment, multivariate analysis for cases identified increased risk of symptoms impacting employment with increasing WHO COVID-19 severity (OR [95%CI] 2.813 [1.194–6.626], p < 0.01) and reduced risk in those receiving statins on admission or who had an elevated CRP (OR [95%CI] 0.28 [0.1–0.778], p < 0.01 and 0.276 [0.099–0.773], p < 0.01 respectively), although the AUROC [95%CI] was only 0.561 [0.467–0.655] [Fig. 5]. For controls, symptoms affecting employment were associated with female sex (OR [95%CI] 2.56 [1.56–5.66], p < 0.01), and the AUROC [95%CI] was 0.714 [0.626–0.802] [Fig. 5].

## Discussion

In this longitudinal case–control study, our objective was to identify the relative proportion of patients with a poor functional outcome in those with and without neurological or psychiatric complications of COVID-19, and to identify clinical risk factors for poor outcomes in both groups. We identified that those who developed a neurological or psychiatric complication of COVID-19 were more likely to have a poor outcome at follow-up at median interval of more than 12 months after hospital discharge, based on impairment of their ADLs and persisting symptoms impacting their employment compared to those hospitalised with COVID-19 alone. Within this group, patients who had developed neuropsychiatric complications were most likely to have impairment in ADLs, symptoms impacting employment, and also an impact on their mental health, in the form of depressive symptoms with PHQ-9 score >5. It is worth noting that patients with neuropsychiatric diagnoses have ADL impairment as a part of DSM-5 diagnostic criteria, and this perhaps might be the reason that they seem to be the worst affected group in the ADL impairment compared to the other groups. The patients with peripheral neurological complications were the next most likely diagnostic group to have both impairment of the ADLs and occupational impact. In addition, patients who had had encephalopathy due to COVID-19 were more likely than COVID-19 controls to have symptoms impacting employment and depression. Comparing the case and control groups overall, there were no significant differences in key demographic features, however, although the case group was more likely to have had mild to moderate COVID-19 respiratory illness on admission, they were more likely to require ventilation and critical care support during the admission.

Further univariate analysis of the case group identified current or previous smoking as a risk factor for impairment of ADLs, and use of angiotensin-inhibiting drugs on admission was associated with reduced risk. On analysis of the risk factors on multivariate logistic regression among the case group, patients aged more than 50 years old, female sex, and hypertension were more likely to have poor functional outcomes. In addition, patients who were on ACE-inhibiting medication or statins on admission had a better functional outcome among the overall case group. Similar to the case group, females in the control group were at higher risk of having poor functional outcomes regarding ADLs and occupational impact on multivariate analyses. This constitutes an important finding for both case and control groups and may reflect the compound effects of perimenopausal, post-menopausal symptoms and challenges, including fatigue and sleep disturbance. The potentially ‘protective’ effect of the management of vascular risk factors on admission (i.e. receiving statins or angiotensin inhibitors) may represent an epiphenomena for other factors, such as patients more actively engaged with healthcare systems, or socioeconomic factors. Nevertheless, this finding in both cases and controls warrants further study as this may represent potentially modifiable risk factors for future epidemic and pandemic infections that impact endothelial biology. The potential mechanisms by which the risk and ‘protective’ factors may be associated with the complications and disabilities require future research to determine the underlying pathophysiological mechanisms.

A broad spectrum of neurological or psychiatric complications of COVID-19 has been recognised in several early studies since the beginning of the pandemic^1,3,7,18^. Several reports have demonstrated that patients with neurological and psychiatric complications associated with COVID-19 are likely to have worse clinical and functional outcomes while in the hospital and post-discharge^6,7,18–24^. This is consistent with the findings of our study, although the longitudinal follow-up of our patients demonstrates impairments are often still present over 12 months after discharge, and that they have demonstrable impacts on key factors for both independence and quality of life. Moreover, this study identified demographic and clinical features associated with better or worse functional outcomes, particularly female sex, and older age versus management of vascular risk factors respectively. Compared to the patients without neurological or psychiatric complications of COVID-19, the cases were at higher risk of requiring critical care admission in our study, which is similar to previous cohort studies^19,24^. This could also be one of the contributing factors for poor functional outcomes in the case group in our cohort, although univariate analysis within the case group did not show any significant differences in the outcome for those requiring critical care admission.

Cohort studies have reported that some patients with neurological complications associated with COVID-19 may have a favourable long-term outcome with regards to symptoms and that the incidence of neurological complications declined over the course of the pandemic, in part due to changes in treatment, including dexamethasone and remdesivir, and with changes in the predominant circulating SARS-CoV-2 variant^25,26^. However, in our cohort, the outcome measure was based specifically on the impairment of ADLs, rather than persisting symptoms and this could be an explanation for these poor functional outcomes.

The participants in our cohort reported having persistent symptoms that were having an impact on their employment, which was more frequent in the case group compared to the controls over a median interval of 13–16 months after discharge, which has potentially significant health and economic implications. Therefore, COVID-19 patients, particularly those who had neurological or psychiatric complications, and more so specifically for those aged > 50 years old, females, and those with vascular risk factors like hypertension, could be targeted for multidisciplinary team support by the healthcare professionals, occupational health, and social care workers post-discharge, to reduce potential long-term occupational impacts.

Mental health outcomes were not significantly different in both groups overall. This is potentially due to the impacts on both groups of COVID-19, hospitalisation, and the broader impacts of the pandemic. However, among the case group the rate of depression was 46% compared to 34% in other groups, as determined by PHQ-9 score >5. Although well established, the PHQ-9 lacks the precision of a diagnostic interview and, type 2 error cannot be excluded. This is a potential confounder as depression has a strong, established negative effect on health-related quality of life.

In conclusion, this study suggests that the development of neurological or psychiatric complications from COVID-19 may identify a highly vulnerable patient group who have a greater risk of morbidities leading to poor functional outcomes and a significant impact on their occupation. Consideration should be given to early recognition and improved access to rehabilitation measures for these patients, to reduce the impact on their daily functional status and employment in the longer term. Further research is needed to determine if the management of vascular risk factors (e.g., statins and angiotensin inhibitors) is associated with improved longer-term outcomes in other cohorts and may represent potentially modifiable factors in future epidemic or pandemic infections.

**Table 4:** Univariate and multivariate analysis of the case group—ADL Impairment.

Risk Factors (NA = not available data)	ADL Impaired (n = 199) n(row %)	Not ADL impaired (n = 90) n(row %)	All (n = 289)	Univariate analysis Odds ratio (95% confidence interval), p value	Multivariate analysis Odds ratio (95% confidence interval), p value
Female Male	*80 (72)* *119 (66)*	*31 (28)* *59 (33)*	**111** **178**	1.28 (0.76 – 2.13), 0.36	5.43 (1.73—16.95), 0.003**
Age > 50 Age < 50	*134 (69)* *65 (67)*	*58 (31)* *32 (33)*	**192** **97**	1.27 (0.75 – 2.18), 0.41	3.11 (1.17—8.26), 0.02*
Smoker Non-Smoker (NA = 23)	*79 (74)* *97 (61)*	*28 (26)* *62 (39)*	**107** **159**	1.80 (1.05 – 3.14), 0.03*	1.47 (0.56—3.83), 0.42
Ethnicity – White Ethnicity – Non-White	*155 (71)* *44 (62)*	*63 (29)* *27 (38)*	**218** **71**	1.51 (0.87 – 2.65), 0.18	2.07 (0.68—6.28), 0.19
Frailty Score > 2 Frailty Score < 2 (NA = 7)	*60 (75)* *136 (68)*	*20 (25)* *66 (32)*	**80** **202**	1.46 (0.81 – 2.62). 0.25	-
Hypertension Normotension (NA = 10)	*65 (67)* *128 (71)*	*33 (33)* *52 (29)*	**98** **180**	1.20 (0.67 – 2.11). 0.56	3.66 (1.06—12.68), 0.04*
Diabetes No Diabetes (NA = 11)	*24 (65)* *168 (70)*	*13 (35)* *72 (30)*	**37** **240**	0.79 (0.38—1.58), 0.56	0.58 (0.17—1.94), 0.38
Renal disease No Renal disease (NA = 11)	*4 (50)* *189 (70)*	*4(50)* *80 (30)*	**8** **269**	0.42 (0.12 – 1.49), 0.25	-
Dyslipidaemia No Dyslipidaemia (NA = 18)	*24 (63)* *164 (70)*	*14 (37)* *69 (30)*	**38** **233**	0.72 (0.36 – 1.52), 0.45	3.44 (0.71—16.64), 0.12
Stroke No Stroke (NA = 11)	*5 (63)* *187 (70)*	*3 (37)* *82 (30)*	**8** **269**	0.73 (0.19 – 2.82), 0.70	-
COVID Vaccine No Vaccine (NA = 8)	*173 (70)* *24 (71)*	*74 (30)* *10 (29)*	**247** **34**	0.97 (0.46—2.17), > 0.9	-
ACEI/ARB No ACEI/ARB (NA = 12)	*22 (55)* *172 (73)*	*18 (45)* *65 (27)*	**40** **237**	0.46 (0.23 – 0.94), 0.03*	0.17 (0.03—0.84), 0.03*
Statins No Statins (NA = 18)	*31 (62)* *157 (71)*	*19 (38)* *63 (29)*	**50** **220**	0.65 (0.35 – 1.26), 0.23	0.09 (0.02—0.36), 0.0006****
Steroids No Steroids (NA = 27)	*33 (80)* *148 (67)*	*8 (20)* *72 (33)*	**41** **220**	2.00 (0.89 – 4.31), 0.10	-
O2 on Admission No O2 on admission (NA = 11)	*99 (73)* *94 (66)*	*36 (27)* *48 (34)*	**135** **142**	1.40 (0.84 – 2.32), 0.24	0.28 (0.04 – 1.75), 0.17
O2 Peak Admission No O2 Mid admission (NA = 43)	*92 (70)* *76 (68)*	*40 (30)* *36 (32)*	**132** **112**	1.09 (0.63—1.86), 0.78	2.36 (0.35—15.75), 0.38
CRP > 5 CRP < 5 (NA = 96)	*110 (71)* *28 (72)*	*44 (29)* *11 (28)*	**154** **39**	0.98 (0.45—2.18), > 0.9	1.04 (0.22—4.87), 0.96
Critical Care Admission No Admission (NA = 14)	*67 (73)* *127 (69)*	*25 (27)* *57 (31)*	**92** **184**	1.20 (0.70 – 2.09), 0.57	1.82 (0.61—5.42), 0.28

NA: ADL: activities of daily living, Not Available (data), CRP: C-reactive protein, ACEI: Angiotensin converting enzyme inhibitors, ARB: Angiotensin receptor blockers. O2: Oxygen. Risk factors applied to the multivariable regressions include gender, Age > 50, Smoking status, Ethnicity, Hypertension, Diabetes, Dyslipidaemia, ACEI/ARB, Statins, Oxygen requirement on admission and mid of admission, CRP > 5, Critical care admission.

### Strengths and limitations of the study

This is a multicentre longitudinal case–control study of a large number of participants, who were appropriately matched at group level to have similar baseline characteristics, epoch of hospitalisation, and time from discharge to follow-up assessment. The outcome measures were intentionally self-reported, based on the patient’s own lived experience and perception of their independence for ADLs and symptoms impacting employment following discharge. Data collection, case classification, and interpretation of analyses were performed by a multidisciplinary team, including specialists in neurology, stroke, psychiatry, and neurointensive care to optimise diagnostic accuracy for the selected group of participants.

However, of 651 patients recruited, follow-up data for assessment of functional outcomes was only available for 484, which may risk selection bias either to those most or least affected, as they may be more concerned about their symptoms or more able to complete the follow-up assessment respectively. Secondly, due to the proportion without a completed mRS score, an adjusted ADL scoring tool based on the data availability was used in the study. Although this is not an externally validated tool, it was adapted based on the questions from approved ADL outcome measuring tools (ALSFRS, UPDRS) with ‘acceptable’ internal validity based on cronbach’s alpha 0.791 and 0.777. In addition, this adjusted ADL tool was aligned with the mRS score, with anyone having normal ADLs were grouped into mRS scores 0–1, a good functional outcome, and those with impaired ADLs were grouped into mRS scores 2–4, a poor functional outcome. Thirdly, functional outcomes following neurological complications with COVID-19, particularly stroke, encephalopathy, encephalitis can be worse compared to those without COVID-19 infection, however, in the interest of statistical power, those with complications (i.e. cases) were grouped for analysis relative to those without the complications (i.e. controls) in our study ^27,28^ Nevertheless, subgroup analysis is provided in Table 3 and described in the results section. Given the number of patients with specific diagnoses, we could not make definite conclusions as to whether one condition had a worse outcome, as this would need a further large-scale study on a bigger population with each condition following exposure to COVID-19. Fourthly, among the risk factors, a formal socio-economic status was not used apart from education status and employment, acknowledging this could be a significant risk factor with health behaviours or access problems which may be associated with poor functional outcomes. Finally, the duration of the study from March 2020 to July 2022 means patients were recruited from at least four waves of the pandemic in the UK by different SARS-CoV-2 variants, but these data were not further analysed, as there was no available data of specific variants for individual participants in the cohort.

## Conclusions

In this large multi-centre case–control study, we identified that patients with neurological or psychiatric complications associated with COVID-19 were at higher risk of having impairment in their activities of daily living compared to general hospitalised COVID-19 patients and are more prone to have persisting symptoms affecting their employment even > 12 months after discharge from hospital. Being female, aged more than 50 years old, and having hypertension were associated with a poor functional outcome, and being on angiotensin inhibitors or statins was associated with good functional outcomes. These findings have implications for the importance of identifying these patients at risk of poor functional outcome, for engagement with a multidisciplinary approach to rehabilitation and support to address the longer-term morbidities and also for future epidemic or pandemic infections.

## Supplementary Information

Supplementary Information.

## Data Availability

Data are available on reasonable request through the Data Access Committee of the national NIHR BioResource [https://bioresource.nihr.ac.uk, email: dac@bioresource.nihr.ac.uk].
