# Supplementary material for: Patients with neurological or psychiatric complications of COVID-19 have worse long-term functional outcomes: COVID-CNS—A multicentre case–control study
Source: Sci Rep. 2025 Jan 27;15:3443. doi: 10.1038/s41598-024-80833-0 (PMC11772688; doi:10.1038/s41598-024-80833-0)
Supplement: Supplementary file 1 — Supplementary Information. [file 41598_2024_80833_MOESM1_ESM.pdf]

Supplementary Data

Patients with neurological or psychiatric complications of COVID-19 have worse long-term functional outcomes: COVID-CNS- A multicentre case-control study.

*Shil RSK, Seed A, Egbe Franklyn, N, Sargent B, Wood GK, Huang Y, Dodd KC, Lilleker JB, Pollack TA, Defres S, Jenkins TM, Davies NWS, Cousins DA, Zandi MS, Jackson TA, Benjamin LA, Easton A, Solomon T, Bradley JR, Chinnery PF, Smith CJ, Nicholson TR, Carson A, Thomas RH, Ellul MA, Wood NW, Breen G, (on behalf of the COVID-CNS Study Group) Michael BD.

Authors:

- *Shil, Rajish SK[1,2,3,6], First Author, ORCID: 0000-0001-9100-6114, rajishsk@liverpool.ac.uk
- Seed, Adam[3], Co-Author, ORCID: 0009-0008-3314-471X
- Egbe Franklyn, Nkongho[1], Co-Author, ORCID: 0000-0002-9547-4609
- Sargent, Brendan F[1,4,5], Co-Author, ORCID: 0000-0003-2262-7755
- Wood, Greta K[1,3], Co-Author, ORCID: 0000-0001-6098-2331
- Huang, Yun[1,2,6], Co-Author, ORCID: 0000-0001-7843-9126
- Dodd, Katherine C[7], Co-Author, ORCID: 0000-0002-0491-7030
- Lilleker, James B[7,8], Co-Author, ORCID: 0000-0002-9230-4137
- Pollak, Thomas A[9,10], Co-Author, ORCID: 0000-0002-6171-0810
- Defres, Sylviane[1,2], Co-Author, ORCID: 0000-0002-4729-7505
- Jenkins, Thomas M[11-14], Co-Author, ORCID: 0000-0003-0342-7184
- Davies, Nicholas WS[15], Co-Author, ORCID: 0000-0003-4095-4549
- Cousins, David A[16], Co-Author, ORCID: 0000-0001-5682-7507
- Zandi, Michael S[17], Co-Author, ORCID: 0000-0002-9612-9401
- Jackson, Thomas A[18], Co-Author, ORCID: 0000-0001-6320-9600
- Benjamin, Laura A[6,19], Co-Author, ORCID: 0000-0002-9685-1664
- Easton, Ava[1,6,20], Co-Author, ORCID: 0000-0002-1739-2915
- Solomon, Tom[1,2,6,21], Co-Author, ORCID: 0000-0001-7266-6547
- Bradley, John R[22], Co-Author, ORCID: 0000-0002-7774-8805
- Chinnery, Patrick F[23,24], Co-Author, ORCID: 0000-0002-7065-6617
- Smith, Craig J[7,25,26], Co-Author, ORCID: 0000-0002-4828-2700
- Nicholson, Tim R[9,10], Co-Author, ORCID: 0000-0002-4367-3956
- Carson, Alan[27], Co-Author, ORCID: 0000-0002-7425-0964
- Thomas, Rhys H[28,29,30], Co-Author, ORCID: 0000-0003-2062-8623
- Ellul, Mark Alexander[1,2,6], Co-Author, ORCID: 0000-0002-6115-8245
- Wood, Nicholas W[31], Co-Author, ORCID: 0000-0002-9500-3348
- Breen, Gerome[1], Co-Author, ORCID: 0000-0003-2053-1792
- Michael, Benedict Daniel[1,2,6,20] (On Behalf of the COVID-CNS Study Group), ORCID: 0000-0002-8693-8926

1. Clinical Infection, Microbiology & Immunology, Institute of Infection, Veterinary and Ecological Sciences, University of Liverpool, Liverpool, United Kingdom (UK)
2. Department of Neurology, Walton Centre of Neurosurgery and Neurology, Liverpool, UK

3. Liverpool University Hospitals NHS Foundation Trust, Liverpool, UK
4. Nuffield Department of Clinical Neurosciences, University of Oxford, John Radcliffe Hospital, Oxford, OX3 9DU, UK
5. Department of Psychiatry, University of Oxford, Oxford, OX3 7JX, UK
6. National Institute for Health and Care Research Health Protection Research Unit in Emerging and Zoonotic Infections, Institute of Infection, Veterinary and Ecological Sciences, University of Liverpool, Liverpool, UK
7. Manchester Centre for Clinical Neurosciences, Northern Care alliance NHS Foundation Trust, Manchester Academic Health Science Centre, Salford, M6 8HD, UK
8. Division of Musculoskeletal & Dermatological Sciences, School of Biological Sciences, Faculty of Biology, Medicine and Health, School of Medical Sciences, University of Manchester, Manchester, UK
9. Department of Psychosis Studies, Institute of Psychiatry, Psychology and Neuroscience, King’s College London, London, SE5 4AF, UK
10. South London and Maudsley NHS Foundation Trust, London, UK
11. Curtin University, Kent Street, Bentley, Perth, 6102, Western Australia
12. Sheffield Institute for Translational Neuroscience, 385a Glossop Road, Sheffield, UK
13. Department of Neurology, Joondalup Healthcare Campus, 60 Shenton Avenue, Joondalup, 6027, Perth, Western Australia
14. Midland St John of God Hospital, 1 Clayton Street, Midland, 6056, Perth, Western Australia
15. Department of Neurology, Charing Cross Hospital, London, UK
16. Translational and Clinical Research Institute, Faculty of Medical Sciences, Newcastle University, Campus for Ageing and Vitality, Newcastle upon Tyne, NE4 5PL, UK
17. UCL Queen Square Institute of Neurology, University College London, London, WC1N 3BG, UK
18. MRC-Versus Arthritis Centre for Musculoskeletal Ageing Research, Institute of Inflammation and Ageing, University of Birmingham, Birmingham, UK
19. Laboratory of Molecular and Cell Biology, University College London, Gower St, King's Cross, London, London, WC1E 6BT, UK
20. Encephalitis International, Malton, UK
21. The Pandemic Institute, The Spine, Liverpool, L7 3FA, UK
22. NIHR BioResource, Cambridge University Hospitals NHS Foundation, Cambridge Biomedical Campus, Cambridge, UK
23. Department of Clinical Neurosciences, University of Cambridge, UK.
24. MRC Mitochondrial Biology Unit, University of Cambridge, UK
25. Geoffrey Jefferson Brain Research Centre, Clinical Sciences Building, Northern Care Alliance NHS Foundation Trust, Salford, M6 8FJ, UK
26. Division of Cardiovascular Sciences, Faculty of Biology, Medicine and Health, School of Medical Sciences, University of Manchester, Manchester, UK
27. Centre for Clinical Brain Sciences, University of Edinburgh, Edinburgh, EH16 4SB, UK
28. Translational and Clinical Research, Newcastle University, Newcastle, NE1 7RU, UK
29. Wellcome Centre for Mitochondrial Research, Newcastle University, Newcastle, NE2 4HH, UK
30. Department of Neurology, Royal Victoria Infirmary, Newcastle, NE1 4LP, UK
31. Department of Molecular Neuroscience, UCL Institute of Neurology, London, UK

Table s1: Clinical Case definitions of the patients identified as cases as per previously agreed criteria [1].

Definitions	Criteria
**Cerebrovascular Events**	Symptoms, signs, and/or neuroimaging consistent with a transient ischaemic attack, ischaemic or haemorrhagic stroke, intracranial venous thrombosis, posterior reversible encephalopathy syndrome, or giant cell arteritis.
**Central inflammatory conditions**	Signs involving the CNS, with evidence of meningeal, parenchymal, or vascular inflammation (CSF white cell count > 4/mm³, and/or protein > 0.45 g/dl, and/or neuroimaging consistent inflammation and/or demyelination).
**Peripheral neuropathies**	Cases involving the peripheral nervous system and categorized as inflammatory and non-inflammatory, based on the reported diagnosis and whether inflammation is the sole recognized pathophysiological cause of this diagnosis.
**Encephalopathy/Delirium DSM-5 and the Ten Societies position statement**	New-onset disturbance in attention, awareness, and cognition, developing over hours or days, with some fluctuation, not in the context of a severely reduced level of arousal, such as coma, and not secondary to medication or substance misuse
	Encephalopathy attributable to fever/sepsis, and/or hypoxia–ischaemia. Therefore, severe encephalopathy was defined as those with a severely reduced level of arousal (a Glasgow coma score ≤13/15 and/or seizures).
**Neuropsychiatric Complications**	Cases fulfilling DSM-5 criteria for anxiety, depression, psychosis, post-traumatic stress disorder, and dementia
**Others**	Headaches, Fatigue, anosmia, ageusia, autonomic dysfunction, seizures, movement disorders, cerebral hypoxic injury, speech, and sensory disturbances, generalised weakness.
**Unclassified**	Patients are identified as ‘Cases’ by the clinicians completing the nCRF forms, patients with diagnosis of uncertainty and/or lies between two or more categories of the above clinical case definitions.

Abbreviations: CNS: Central nervous system, CSF: Cerebrospinal fluid, DSM-5: Diagnostic and statistical manual of mental disorders, fifth edition, nCRF: neuro case record form.

Figure s1: ADL Scoring tool for functional outcomes

ADL Scoring Tool
Slight 1-4, Mild 5-8, moderate 9-13, severe>13

	0	1	2	3	4
	Normal	Slight	Mild	Moderate	Severe
Hobbies					
Reading/writing					
Getting out (bed/car/chair)					
Balance in walking					
Personal Hygiene					
**Dichotomise the results - for Binary logistic regression model**					
**Normal (Score 0) = Normal ADL = Good Functional outcome (Equivalent to mRS = 0-1)**					
**Slight to severe (Score 1-20) = Abnormal ADL = Poor functional outcome (Equivalent to mRS = 2-4)**					

Abbreviations: ADL: Activities of daily living, mRS: modified Rankin Score.

ADL Questions used for the tool from the UPDRS

The items used are as below

1. updrs.trouble_reading_handwriting_people
2. updrs.hobbies_trouble_things_past
3. updrs.balance_walking_past_week
4. updrs.personal_hygiene_hair_slow
5. updrs.deep_chair_car_seat, updrs.usual_day_feet_floor, updrs.trouble_turning_bed_past

Figure s2: Cronbach's alpha internal validity of the ADL scoring tool

Cases

	Column 1	285	424	1.487719	0.842278		
	Column 2	285	623	2.185965	2.032197		
	Column 3	285	531	1.863158	1.308673		
	Column 4	285	422	1.480702	0.820929		
	Column 5	285	471	1.652632	0.945812		

ANOVA						
Source of Varia	*SS*	*df*	*MS*	*F*	*P-value*	*F crit*
Rows	921.7993	284	3.245772	4.801231	4.29E-80	1.162281
Columns	100.4309	4	25.10772	37.13999	3.21E-29	2.379764
Error	767.9691	1136	0.676029			
Total	1790.199	1424				
	Cronbach	0.79172				

Controls

	Column 1	195	229	1.174359	0.278721		
	Column 2	195	306	1.569231	0.999048		
	Column 3	195	285	1.461538	0.734338		
	Column 4	195	238	1.220513	0.286175		
	Column 5	195	284	1.45641	0.568966		

ANOVA						
Source of Varia	*SS*	*df*	*MS*	*F*	*P-value*	*F crit*
Rows	294.0574	194	1.51576	4.486195	1.41E-50	1.198429
Columns	22.61128	4	5.652821	16.73065	3.66E-13	2.383407
Error	262.1887	776	0.337872			
Total	578.8574	974				
	Cronbach	0.777094				

Figure s3: Study population Age and gender matching

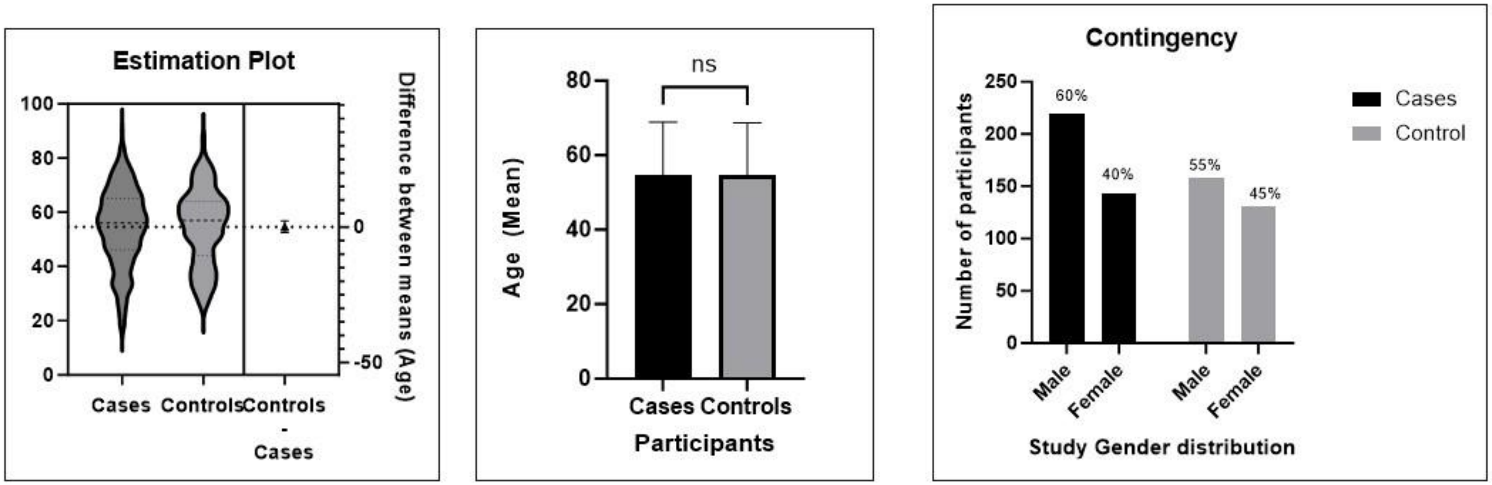

Two- tailed, Unpaired t-test, p value = 0.909,
Not significantly different

Fisher's Exact test, p value = 0.151
Not significantly different

Table 1. Case vs control- Demographics

Demographics	Cases Combined (n=362)	Controls (n=289)	Odds Ratio (95% CI), p value	Cases Cerebrovascular (n=80)	Cases Encephalopathy (n=57)	Cases Inflammatory (n=44)	Cases Neuropsychiatric (n=49)	Cases Others (n=75)	Cases Peripheral (n=51)	Cases Unclassified (n=6)
**Age**										
Mean	54.33 (17-90)	54.68 (24-88)	P = 0.91	55.41 (20-90)	56.15 (18-83)	47.84 (20-72)	52.29 (25-82)	56.84 (17-82)	55.03 (22-77)	63 (29-84)
Median (IQR)	56 (46-65)	57 (44-64)		59 (45.25-66)	58 (47.5-66.5)	48 (35.5-57.5)	52 (43-59.5)	65 (48-58)	55 (48-64)	62.5 (50-83.25)
Age <50	123 (33.9%)	91 (31.3%)	1.12 (0.81-1.56), 0.50	24 (30.0%)	17 (29.8%)	23 (52.2%)	20 (40.8%)	21 (28.0%)	17 (33.3%)	1 (17.0%)
Age >50	239 (66.0%)	198 (68.7%)	0.89 (0.64-1.23), 0.50	56 (70.0%)	40 (70.2%)	21 (47.8%)	29 (59.2%)	54 (72.0%)	34 (66.6%)	5 (83.0%)
Male	219 (60.5%)	158 (54.6%)	1.27 (0.92-1.73), 0.15	50 (62.5%)	36 (62.2%)	27 (61.4%)	27 (55.1%)	45 (60.0%)	32 (62.7%)	2 (33.3%)
Female	143 (39.5%)	131 (45.3%)	0.79 (0.58-1.08), 0.15	30 (37.5%)	21 (36.8%)	17 (38.6%)	22 (44.9%)	30 (40.0%)	19 (37.3%)	4 (66.7%)
White	256 (70.7%)	210 (72.7%)	0.82 (0.56-1.17), 0.28	56 (70.0%)	38 (66.6%)	31 (70.5%)	34 (69.4%)	52 (69.3%)	41 (80.4%)	4 (66.7%)
Non white	97 (26.8%)	65 (22.5%)	1.22 (0.86-1.77), 0.28	21 (26.3%)	17 (29.8%)	11 (25.0%)	15 (30.6%)	21 (28.0%)	10 (19.6%)	2 (33.3%)
Black	39 (10.8%)	23 (7.9%)	1.39 (0.79-2.40), 0.24	10 (12.5%)	6 (10.5%)	3 (6.8%)	4 (8.2%)	12 (16.0%)	3 (5.9%)	1 (16.7%)
Asian	15 (4.1%)	12 (4.2%)	1.02 (0.46-2.24), 0.95	3 (3.75%)	3 (5.3%)	2 (4.5%)	2 (4.1%)	0	4 (7.8%)	0 (0.0%)
Others	30 (8.3%)	20 (6.9%)	1.23 (0.69-2.23), 0.49	6 (7.5%)	5 (8.8%)	4 (9.1%)	8 (16.3%)	4 (5.3%)	3 (5.9%)	1 (16.7%)
Mixed	13 (3.6%)	10 (3.5%)	1.07 (0.46-2.43), 0.89	2 (2.5%)	3 (5.3%)	2 (4.5%)	1 (2.0%)	5 (6.7%)	0	0
NA	10 (2.7%)	14 (4.8%)		4 (5.0%)	2 (3.5%)	2 (4.5%)	0	2 (2.7%)	0	0
University/college	122 (33.7%)	107 (37.0%)	0.72 (0.51-1.0), 0.07	27 (33.8%)	13 (22.8%)	17 (38.6%)	22 (44.9%)	27 (36.0%)	16 (31.4%)	0
School/others	200 (55.2%)	126 (43.6%)	1.39 (0.99-1.95), 0.07	39 (48.8%)	38 (66.7%)	22 (50.0%)	27 (55.1%)	38 (50.7%)	30 (58.8%)	6 (100%)
Prefer not to say	4 (1.1%)	3 (1.0%)		0	0	2 (4.5%)	0	1 (1.3%)	1 (2.0%)	0
NA	36 (9.9%)	53 (18.3%)		14 (17.5%)	6 (10.5%)	3 (6.8%)	0	9 (12.0%)	4 (7.8%)	0
Employed/Self	201 (55.5%)	140 (48.4%)	1.02 (0.69-1.54), 0.92	35 (43.75%)	27 (47.4%)	25 (56.8%)	34 (69.4%)	42 (56.0%)	36 (70.6%)	2 (33.3%)
Retired/unemployed	76 (21.0%)	54 (18.6%)	0.98 (0.65-1.47), 0.92	21 (26.25%)	12 (21.1%)	9 (20.5%)	8 (16.3%)	15 (20.0%)	7 (13.7%)	4 (66.7%)
NA	85 (23.5%)	95 (32.9%)		24 (30.0%)	18 (31.6%)	10 (22.8%)	7 (14.3%)	18 (24.0%)	8 (15.7%)	0
Active Smoker	18 (5.0%)	16 (5.5%)	0.97 (0.47-1.90), >0.99	6 (7.5%)	1 (1.8%)	5 (11.4%)	3 (6.1%)	1 (1.3%)	2 (3.9%)	0
Ex-Smoker	119 (32.9%)	78 (27.0%)	1.32 (0.93-1.86), 0.11	27 (33.7%)	23 (40.4%)	9 (20.5%)	12 (24.5%)	27 (36.0%)	17 (33.3%)	4 (66.7%)
Non-Smoker	225 (62.1%)	195 (67.5%)		47 (58.8%)	33 (57.8%)	30 (68.1%)	34 (69.4%)	47 (62.7%)	32 (62.8%)	2 (33.3%)

Abbreviations: IQR: Interquartile range, NA: Not Available (data), CI: Confidence intervals

Table 2. Case vs control- Clinical features

Risk Factors	Cases Combined (n=362) (%)	Controls (n=289) (%)	Odd's Ratio (95% CI), p value (Excluding NA)	Cases Cerebrovascular (n=80)	Cases Encephalopathy (n=57)	Cases Inflammatory (n=44)	Cases Neuropsychiatric (n=49)	Cases Others (n=75)	Cases Peripheral (n=51)	Cases Unclassified (n=6)
Hypertension	126 (34.8%)	87 (30.1%)	1.24 (0.894 - 1.73), 0.21	33 (41.3%)	25 (43.9%)	12 (27.3%)	14 (28.6%)	23 (30.7%)	15 (29.4%)	4 (66.7%)
Diabetes	56 (15.5%)	43 (14.9%)	1.05 (0.680 – 1.60), 0.91	9 (11.3%)	15 (26.3%)	6 (13.6%)	4 (8.2%)	17 (22.7%)	5 (9.8%)	0 (0.0%)
Dyslipidaemia	48 (13.3%)	39 (13.5%)	0.98 (0.622 – 1.52), >0.99	18 (22.5%)	8 (14.0%)	2 (4.5%)	4 (8.2%)	7 (9.3%)	8 (15.7%)	1 (16.7%)
Renal Failure	12 (3.3%)	15 (5.2%)	0.63 (0.289 – 1.39), 0.24	1 (1.3%)	3 (5.3%)	0	0	5 (6.6%)	3 (5.9%)	0
Previous Stroke	13 (3.6%)	2 (0.1%)	5.35 (1.397 – 23.96), 0.02*	7 (8.8%)	2 (3.5%)	1 (2.3%)	0	3 (4.0%)	0	0
Obesity/high BMI	11 (3.0%)	15 (5.2%)	0.57 (0.25 – 1.29), 0.23	2 (2.5%)	3 (5.3%)	1 (2.3%)	1 (2.0%)	2 (2.7%)	1 (2.0%)	1 (16.7%)
Psychiatry History	43 (11.9%)	25 (8.7%)	1.42 (0.85 – 2.42), 0.19	5 (6.3%)	5 (8.8%)	3 (6.8%)	9 (18.3%)	16 (21.3%)	5 (9.8%)	0
No Psychiatric History	319 (88.1%)	264 (91.3%)	2.23 (0.69 – 6.45), 0.19	75 (93.7%)	52 (91.2%)	41 (93.2%)	40 (81.7%)	59 (78.7%)	46 (90.2%)	6 (100%)
Neurological History	11 (3.0%)	4 (1.4%)		4 (5.0%)	2 (3.5%)	1 (2.3%)	1 (2.0%)	3 (4.0%)	0	0
No Neurological History	351 (97.0%)	285 (98.6%)		76 (95.0%)	55 (96.5%)	43 (97.7%)	48 (98.0%)	72 (96.0%)	51 (100%)	6 (100%)
COVID Vaccination	251 (69.3%)	171 (59.2%)	1.14 (0.62 – 2.10), 0.75	50 (62.5%)	34 (59.6%)	31 (70.5%)	39 (79.6%)	50 (66.7%)	42 (82.4%)	5 (83.3%)
Not answered	84 (23.2)	97 (33.6%)		23 (28.8%)	16 (28.1%)	9 (20.5%)	8 (16.3%)	21 (28.0%)	7 (13.7%)	0
No vaccination	27 (7.5%)	21 (7.2%)		7 (8.7%)	7 (12.3%)	4 (9.0%)	2 (4.1%)	4 (5.3%)	2 (3.9%)	1 (16.7%)
ACEI/ARB	61 (16.9%)	43 (14.9%)	1.16 (0.77 – 1.75), 0.52	16 (20.0%)	13 (22.8%)	6 (13.6%)	9 (18.3%)	10 (13.3%)	5 (9.8%)	2 (33.3%)
Statins	68 (18.8%)	56 (19.4%)	0.96 (0.64 – 1.42), 0.92	18 (22.5%)	19 (33.3%)	3 (6.8%)	7 (14.3%)	14 (18.7%)	6 (11.8%)	1 (16.7%)
Steroids	52 (14.4%)	56 (19.4%)	0.69 (0.47 – 1.04), 0.09	7 (8.8%)	9 (15.8%)	4 (9.1%)	8 (16.3%)	13 (17.3%)	9 (17.6%)	2 (33.3%)
COVID-19 severity on admission										
Moderate (no oxygen)	158 (43.6%)	101 (34.9%)	1.65 (1.19 – 2.28), 0.002**	49 (61.2%)	13 (22.8%)	31 (70.5%)	22 (44.9%)	27 (36.0%)	16 (31.4%)	0
Severe (oxygen)	90 (24.9%)	139 (48.1%)	0.39 (0.28 – 0.54), <0.0001****	15 (18.7%)	18 (31.6%)	2 (4.5%)	18 (36.7%)	24 (32.0%)	11 (21.6%)	2 (33.3%)
Critical (NIV/MV)	84 (23.2%)	45 (15.6%)	1.80 (1.21 – 2.70), 0.003**	12 (15.0%)	23 (40.4%)	7 (15.9%)	4 (8.2%)	16 (21.3%)	19 (37.3%)	3 (50.0%)
NA	30 (8.3%)	4 (1.4%)		4 (5.0%)	3 (5.3%)	4 (9.1%)	5 (10.2%)	8 (10.7%)	5 (9.8%)	1 (16.7%)
COVID-19 Worst Severity										
Moderate (no oxygen)	123 (34.0%)	76 (26.3%)	1.83 (1.29 – 2.58), 0.0007***	36 (45.0%)	10 (17.5%)	24 (54.5%)	18 (36.7%)	21 (28.0%)	14 (27.5%)	0
Severe (Oxygen)	64 (17.7%)	125 (43.6%)	0.33 (0.23 – 0.48), <0.0001****	9 (11.2%)	12 (21.1%)	4 (9.1%)	11 (22.4%)	18 (24.0%)	9 (17.6%)	1 (16.7%)
Critical (NIV/MV)	109 (30.1%)	71 (24.5%)	1.65 (1.15 – 2.37), 0.006***	22 (27.5%)	26 (45.6%)	7 (15.9%)	12 (24.5%)	21 (28.0%)	17 (33.3%)	4 (66.7%)
NA	66 (18.2%)	17 (5.9%)		13 (16.2%)	9 (15.8%)	9 (20.5%)	8 (16.3%)	15 (20.0%)	11 (21.6%)	1 (16.7%)
Critical Care Admission										
Yes	118 (32.6%)	52 (18.0%)	2.50 (1.74 – 3.65), <0.0001**	28 (35.0%)	29 (50.9%)	10 (22.7%)	9 (18.4%)	16 (21.3%)	22 (43.1%)	4 (66.7%)
NA	12 (3.3%)	8 (2.8%)		2 (2.5%)	0	2 (4.5%)	3 (6.1%)	3 (4.0%)	1 (2.0%)	1 (16.7%)
Admission CRP										
High (>5)	201 (55.5%)	219 (75.8%)	0.48 (2.09 – 0.81), 0.007***	47 (58.8%)	36 (63.2%)	13 (29.5%)	24 (49.0%)	41 (54.6%)	35 (68.6%)	5 (83.3%)
Normal (<5)	46 (12.7%)	24 (8.3%)		8 (10.0%)	4 (7.0%)	12 (27.3%)	8 (16.3%)	11 (14.7%)	3 (5.9%)	0
NA	115 (31.7%)	47 (16.3%)		25 (31.3%)	17 (29.8%)	19 (43.2%)	17 (34.7%)	23 (30.7%)	13 (25.5%)	1 (16.7%)
Frailty Score >2	109 (30.1%)	73 (25.2%)	1.20 (0.84 – 1.74), 0.35	25 (31.3%)	23 (40.4%)	10 (22.7%)	17 (34.7%)	21 (28.0%)	12 (23.5%)	1 (16.7%)
Not answered	64 (17.7%)	64 (22.1%)		15 (18.8%)	11 (19.3%)	5 (11.4%)	1 (2.0%)	19 (25.3%)	11 (21.5%)	2 (66.7%)

Abbreviations: NA: Not Available (data), CI: Confidence intervals, BMI: Body mass index, ACEI: Angiotensin converting enzyme inhibitors, ARB: Angiotensin receptor blockers, NIV: Non-invasive ventilation, MV: Mechanical ventilation, CRP: C-reactive protein.

Table 3. Controls vs control and individual case definitions - Functional outcomes

Functional Outcome	Controls (n=289)	Cases Combined (n=362) (%), OR (95% CI), p value - excluding NA	Cases Cerebrovascular (n=80), OR [95% CI], p value - excluding NA	Cases Encephalopathy (n=57), OR [95% CI], p value - excluding NA	Cases Inflammatory (n=44), OR [95% CI], p value - excluding NA	Cases Neuropsychiatric (n=49), OR [95% CI], p value - excluding NA	Cases Others (n=75), OR [95% CI], p value - excluding NA	Cases Peripheral (n=51), OR [95% CI], p value- excluding NA	Cases Unclassified (n=6), OR [95% CI], p value- excluding NA
**Age**									
Mean	54.68 (24-88)	54.33 (17-90)	55.41 (20-90)	56.15 (18-83)	47.84 (20-72)	52.29 (25-82)	56.84 (17-82)	55.03 (22-77)	63 (29-84)
Median (IQR)	57 (44-64)	56 (46-65)	59 (45.25-66)	58 (47.5-66.5)	48 (35.5-57.5)	52 (43-59.5)	65 (48-58)	55 (48-64)	62.5 (50-83.25)
**ADL Scoring**									
Normal	94 (32.5%)	90 (24.9%)	21 (26.8%)	17 (29.8%)	12 (27.3%)	12 (24.5%)	18 (24%)	9 (17.6%)	1 (16.7%)
**Abnormal (Score >1)**	**101 (35.0%)**	**199 (55.0%), 2.06 (1.40 - 2.98), <0.0002*** **	**36 (45.0%), 1.59 [0.87-2.95], 0.13**	**27 (47.4%), 1.47 [0.75-2.96], 0.31**	**25 (56.8%), 1.93 [0.93-4.04], 0.10**	**31 (63.3%) 2.40 [1.14-4.85], 0.01***	**40 (53.3%), 2.06 [1.11-3.79], 0.02***	**36 (70.6%), 3.72 [1.75-8.25], 0.00007*** **	**4 (66.7%), 3.72 [0.59-45.98], 0.37**
Slight (1-4)	71 (24.6%)	99 (27.3%)	18 (22.5%)	11 (19.3.8%)	10 (22.7%)	19 (38.8%)	18 (24%)	22 (43.1%)	1 (16.7%)
Mild (5-8)	20 (6.9%)	58 (16.0%)	13 (16.3%)	10 (17.5%)	5 (11.4%)	4 (8.2%)	15 (20%)	9 (17.6%)	2 (33.3%)
Moderate (9-13)	10 (3.5%)	34 (9.4%)	5 (6.0%)	5 (8.7%)	9 (20.4%)	5 (10.2%)	4 (5.3%)	5 (9.8%)	1 (16.7%)
Severe (14-20)	0	8 (2.2%)	0	1 (1.8%)	1 (2.2%)	3 (6.1%)	3 (4.0%)	0	0
NA	94 (32.5%)	73 (20.2%)	23 (28.8%)	13 (22.8%)	7 (15.9%)	6 (12.2%)	17 (22.7%)	6 (11.8%)	1 (16.7%)
**Symptom impact**									
**Yes**	**69 (23.9%)**	**159 (43.9%), 2.53 (1.72 - 3.71), <0.0001**** **	**26 (32.5%), 1.57 [0.88-2.90], 0.16**	**23 (40.4%), 2.45 [1.25-4.72], 0.01***	**18 (40.9%), 3.26 [1.40-7.31], 0.006** **	**30 (61.2%), 4.18 [2.0-8.24], <0.0001**** **	**32 (42.7%), 2.31 [1.27-4.14], 0.008** **	**28 (54.9%), 3.38 [1.66-6.70], 0.0005*** **	**2 (33.3%), 0.90 [0.17-3.97], >0.99**
NA	95 (32.9%)	89 (24.6%)	24 (30.0%)	17 (29.8%)	16 (36.4%)	6 (12.2%)	18 (24.0%)	8 (15.6%)	0
**GAD-7 Score**									
Normal (0-4)	126 (43.6%)	175 (48.3%)	43 (53.6%)	21 (36.8%)	26 (59.1%)	22 (44.9%)	32 (42.7%)	29 (56.8%)	2 (33.3%)
**Abnormal (Score >5)**	**73 (25.2%)**	**117 (32.3%) 1.15 (0.79 - 1.67), 0.51**	**14 (17.5%), 0.56 [0.29-1.07], 0.08**	**22 (38.6%), 1.80 [0.95-3.44], 0.08**	**11 (25.0%), 0.73 [0.33-1.56], 0.46**	**24 (48.9%), 1.88 [0.97-3.52], 0.06**	**27 (36.0%), 1.46 [0.79-2.61], 0.22**	**15 (29.4%), 0.89 [0.45-1.79], 0.86**	**4 (66.7%), 3.45 [0.78-18.38], 0.20**
Mild (5-9)	36 (12.5%)	52 (14.3%)	9 (11.3%)	10 (17.5%)	7 (15.9%)	5 (10.2%)	13 (17.3%)	6 (11.8%)	2 (33.3%)
Moderate (10-14)	28 (9.7%)	41 (11.3%)	3 (3.6%)	8 (14.0%)	4 (9.1%)	11 (22.4%)	11 (14.7%)	4 (7.8%)	0
Severe (>15)	9 (3.1%)	24 (6.6%)	2 (2.5%)	4 (7.5%)	0	8 (16.3%)	3 (4.0%)	5 (9.8%)	2 (33.3%
NA	90 (31.1%)	70 (19.3%)	23 (28.8%)	14 (24.6%)	7 (15.9%)	3 (6.1%)	16 (21.3%)	7 (13.7%)	0
**PHQ-9 Score**									
Normal (0-4)	101 (34.9%)	124 (34.2%)	36 (45.0%)	13 (22.8%)	20 (45.5%)	15 (30.6%)	24 (32.0%)	15 (29.4%)	1 (16.7%)
**Abnormal (Score >5)**	**98 (33.9%)**	**167 (46.1%), 1.39 (0.97 - 1.99), 0.08**	**22 (27.5%), 0.34 [0.17-0.69], 0.002** **	**30 (52.6%), 2.38 [1.16-4.74], 0.01***	**16 (36.3%), 0.82 [0.41-1.71], 0.59**	**30 (61.2%), 2.06 [1.04-3.99], 0.03***	**36 (48.0%), 1.54 [0.88-2.71], 0.15**	**28 (54.9%), 1.92 [0.96-3.77], 0.06**	**5 (83.3%), 5.15 [0.69-61.29], 0.09**
Mild (5-9)	45 (15.6%)	79 (21.8%)	11 (13.8%)	10 (17.5%)	8 (18.2%)	13 (26.5%)	17 (22.7%)	18 (35.2%)	2 (33.3%)
Moderate (10-14)	37 (12.8%)	50 (13.8%)	8 (1.0%)	12 (21.1%)	6 (13.6%)	8 (16.3%)	8 (10.7%)	7 (13.7%)	1 (16.7%)
Mod. Severe (15-19)	9 (3.1%)	21 (5.8%)	1 (1.3%)	6 (10.5%)	0	4 (8.2%)	9 (12.0%)	1 (2.0%)	0
Severe (>20)	7 (2.4%)	17 (4.7%)	2 (2.5%)	2 (3.5%)	2 (4.5%)	5 (10.2%)	2 (2.6%)	2 (3.9%)	2 (33.3%)
NA	90 (31.2%)	71 (19.6%)	22 (27.5%)	14 (24.6%)	8 (1.8%)	4 (8.2%)	15 (20.0%)	8 (15.7%)	0
**Became Unemployed**									
Yes	12 (4.2%)	34 (9.4%)	8 (1.0%)	6 (10.5%)	4 (9.1%)	8 (16.3%)	4 (5.3%)	4 (7.8%)	0
NA	245 (84.8%)	258 (71.3%)	63 (78.8%)	43 (75.4%)	31 (70.5%)	27 (55.1%)	54 (72.0%)	36 (70.6%)	4 (66.7%)
**Time to BLQ**									
Median (IQR) months	16 (5-22)	13 (7-17)	11 (5-14)	13 (7.75-16.25)	8 (5.25-13.75)	15 (10-17)	11 (6.75-17)	13 (7-18)	15 (14.5-21.5)
Median (IQR) Days	487 (152-669)	395 (212-517)	334 (152-425)	395 (235-494)	243 (160-418)	456 (304-213)	334 (205-517)	395 (213-546)	456 (441-654)

Abbreviations: IQR: Interquartile range, NA: Not Available (data), OR: Odds ratio, CI: Confidence intervals, ADL: Activities of daily living, GAD-7: Generalised Anxiety Disorder – 7, PHQ-9: Patient Health Questionnaire – 9, BLQ: Baseline Questionnaire

COVID-CNS Study Group / Consortium

John P. Aggleton[32], Ali M. Alam[1], Ammar Al-Chalabi[33], Christopher M. Allen[34], Jay Amin[35], Cherie Armour[36], Mark R. Baker[37], Suzanne Barrett[38], Neil Basu[39], Rahul Batra[33], Alex Berry[19], Laura Benjamin[19], Richard A. I. Bethlehem[40], Bethan Blackledge[41], Sarah A. Boardman[1], John Bradley[22], David P. Breen[27], Gerome Breen[1], Judith Breuer[42], Matthew Broome[43], Ed Bullmore[23], Matthew Butler[33], Alan Carson[27], Hannah Castell[1], Jonathan Cavanagh[39], Patrick Chinnery[23,24], David Christmas[44], David M. Christmas[58], Jonathan R. I. Coleman[33], Alaistair Coles[58], Ceryce Collie[1], Nadine Cossette[45], David Cousins[16], Colm Cunningham[46], Alastair Darby[47], Anthony S. David[48], Nicholas Davies[15], Sylviane Defres[1,2], Katherine C. Dodd[7], Alex Dregan[33], Eugene Duff[49], Cordelia Dunai[1,6], Ava Easton[1,20], Franklyn N. Egbe[1], Mark A. Ellul[1,2,6], Nikos Evangelou[34], Bethany Facer[50], Peter M. Fernandes[51], Richard Francis[52], Ian Galea[35], Afagh Garjani[34], Lily George[33], Valentina Giunchiglia[53], Kiran Glen[33], Rebecca Gregory[54], Michael Griffiths[47], Victoria Grimbly[1], Alexander Grundmann[35], Savini Gunatilake[55], Shahd H. M. Hamid[2,47], Adam Hampshire[56], Marc Hardwick[35], Jade D. Harris[57], Ewan Harrison[58], Neil A. Harrison[59], Paul J. Harrison[60], Monika Hartmann[33], Peter J. Hellyer[56], Claire Hetherington[1], Orla Hilton[1], Julian Hiscox[47], Eva Maria Hodel[47], Angela E. Holland[61], Matthew Hotopf[9], Yun Huang[1,2,6], Stella Hughes[62], Masud Husain[4], Sarosh Irani[4], Thomas A. Jackson[18], Thomas M. Jenkins[11-14], Peter Jezzard[4], Johan Kallberg Zvrskovec[33], Gursharan Kalsi[63], Simon Keller[50], Nathalie Kingston[22], Sandar Kyaw[64], E. Charles Leek[47], Gabriella Lewis[10], James B. Lilleker[7,8], Michael P. Lunn[42], Claire L. MacIver[65], Daniel Madarshahian[12], Parisa Mansoori[66], Naomi Martin[63], Gavin McDonnell[62], Emily McGlinchey[36], Stephen McKeever[1], Ryan McIlwaine[36], Andrew M. McIntosh[51], David K. Menon[24], Benedict D. Michael[1,2,6,20], Karla L. Miller[4], Dina Monssen[33], Christopher M. Morris[37], Ciaran Mulholland[36], Akshay Nair[33], Edward Needham[23], Virginia Newcombe[58], Nathalie Nicholas[3], Timothy R. Nicholson[9,10], Ronan O'Malley[12], Obioma Orazulume[42], Marlies Ostermann[33], Stella-Maria Paddick[16], Alish Palmos[33], Arvind Patel[39], Sharon Peacock[58], Sophie L. Pendered[1,2], Sarah L. Pett[67,68], Thomas A. Pollak[9,10], Angela Roberts[58], Henry C. Rogers[63], Silvia Rota[33], Rustam Al-Shahi Salman[27], Merna Samuel[47], Brendan F. Sargent[1,4,5], Stephen J. Sawcer[58], Adam W. Seed[3], Scott Semple[51], Pamela J. Shaw[12], Rajish S. K. Shil[1,2,3,6], Adam Sieradzki[71], Bhagteshwar Singh[1,3,6], Craig J. Smith[7,25,26], Jacqueline Smith[71], Stephen M. Smith[4], Tom Solomon[1,2,6,21], Leonie Taams[33], Arina Tamborska[1], John-Paul Taylor[16], Kukatharmini Tharmaratnam[69], Rhys H. Thomas[16], Emma Thomson[39], William Trender[53], Zain-Ul-Abideen Ahmad[63], Jonathan Underwood[65], Rachel Upthegrove[43], Tonny Veenith[18], Annalena Venneri[70], Angela Vincent[4], Daniel J. van Wamelen[56], Guy Williams[23], Steven Williams[56], Sui Hsien Wong[33], Greta K. Wood[1,3,6], Nicholas Wood[31], Michael S. Zandi[17], Fernando Zelaya[10,56].

32. School of Psychology, Cardiff University, Cardiff, UK.
33. King's College London, London, UK.
34. University of Nottingham, Nottingham, UK.
35. University of Southampton, Southampton, UK.
36. Queen's University Belfast, Belfast, UK.
37. Newcastle University, Newcastle, UK.
38. Northern Health and Social Care Trust, Antrim, UK.
39. University of Glasgow, Glasgow, UK.
40. Department of Psychology, University of Cambridge, Cambridge, UK.
41. Salford Royal, Northern Care Alliance NHS Foundation Trust, Manchester, UK.
42. University College London, London, UK.
43. Institute for Mental Health, School of Psychology, University of Birmingham, Birmingham, UK.
44. University of Dundee, Dundee, UK.
45. Royal Infirmary of Edinburgh, NHS Lothian, Edinburgh, UK.
46. School of Biochemistry and Immunology, Trinity Biomedical Sciences Institute, Trinity College Dublin, Dublin, Ireland.

47. University of Liverpool, Liverpool, UK.
48. Department of Psychiatry, Institute of Mental Health, UCL, London, UK.
49. UK Dementia Research Institute, Department of Brain Sciences, Imperial College London, London, UK.
50. Department of Pharmacology and Therapeutics, Institute of Systems, Molecular and Integrative Biology, University of Liverpool, Liverpool, UK.
51. Centre for Clinical Brain Sciences, University of Edinburgh, Edinburgh, UK.
52. The Stroke Association, London, UK.
53. Imperial College London, London, UK.
54. Sheffield Teaching Hospitals NHS Foundation Trust, Sheffield, UK.
55. Royal Stoke University Hospital, Stoke-On-Trent, UK.
56. Department of Neuroimaging, Institute of Psychiatry, Psychology & Neuroscience, King's College London, London, UK.
57. Salford Royal, Northern Care Alliance NHS Foundation Trust, Manchester, UK.
58. University of Cambridge, Cambridge, UK.
59. Cardiff University Brain Research Imaging Centre, School of Medicine, Cardiff University, Cardiff, UK.
60. Department of Psychiatry, Warneford Hospital, University of Oxford, Oxford, UK.
61. Nottingham University Hospital, Nottingham, UK.
62. Belfast Health and Social Care Trust, Belfast, UK.
63. Social, Genetic and Developmental Psychiatry Centre, Institute of Psychiatry, Psychology & Neuroscience, King's College London, London, UK.
64. Institute of Mental Health, Nottingham, UK.
65. Cardiff University, Cardiff, UK.
66. National Institute for Health Research (NIHR) Bioresource, London, UK.
67. MRC Clinical Trials Unit, UCL, London, UK.
68. Institute of Clinical Trials and Methodology, UCL, London, UK.
69. Department of Health Data Science, Institute of Population Health, University of Liverpool, Liverpool, UK.
70. University of Sheffield, Sheffield, UK.
71. COVID-CNS Consortium, Liverpool, UK.
